# Cinobufagin induces peroxisome dysfunction through suppression of the peroxisomal biogenesis factor proteins and interferes with lipid metabolism to affect the growth of liver cancer cells

**DOI:** 10.3389/fphar.2026.1860569

**Published:** 2026-10-16

**Authors:** Jingyi Yang, Wanjun Shao, Congying Yu, Qi Wu, Rufei Xu, Xiaoqian Rong, Ailin Yang

**Affiliations:** 1 Shandong Medical and Pharmaceutical University, Yantai, Shandong, China; 2 Qingdao Central Hospital, University of Health and Rehabilitation Sciences, Qingdao, Shandong, China

**Keywords:** cell proliferation, cinobufagin, lipid metabolism, liver cancer, peroxisome dysfunction

## Abstract

**Background:**

Primary liver cancer is the sixth most commonly diagnosed cancer and the third most common cause of cancer death worldwide. Chansu is made by processing and drying the white serous fluid of the posterior ear and skin glands of *Bufo bufo gargarizans* Cantor or *Bufo melanostictus* Schneider. Chansu is a well-known traditional Chinese medicine (TCM) that is mainly used for detoxification, detumescence, analgesia, the treatment of cancer, and heart failure. Cinobufagin, as a bufadienolide, has been reported as the main antitumor active component of Chansu. However, the molecular mechanisms related to the anticancer effects of cinobufagin still remain elusive.

**Purpose:**

The purpose of this study is to explore whether cinobufagin inhibits the growth of HepG2 and SK-HEP-1 tumor cells by inducing peroxisome dysfunction and interfering with lipid metabolism.

**Methods:**

The protein levels of catalase, ABCD3, PEX5, PEX6, and PEX10 in human hepatoma cells were analyzed by Western blotting. The effects of cinobufagin on the viability of HepG2 and SK-HEP-1 cells were detected by cell counting kit-8 (CCK-8) assay and clone formation assay. Small interfering RNA (siRNA) transfection was used to explore whether inhibition of PEX could enhance the inhibitory effect of cinobufagin on cell growth of HepG2 and SK-HEP-1 tumor cells. The effects of cinobufagin on lipid metabolism were analyzed by untargeted metabolomics. The *in vivo* antitumor effects of cinobufagin were verified by animal studies.

**Results:**

Cinobufagin causes peroxisome dysfunction in HepG2 and SK-HEP-1 tumor cells. From a mechanistic point of view, we reveal that cinobufagin downregulates the expression of PEX5, PEX10, and PEX6. In addition, through RNA interference, cinobufagin partially blocks the growth of HepG2 and SK-HEP-1 tumor cells and restrains the expression of lipid metabolism-related proteins *via* downregulating the expression of PEX5 and PEX10. Subsequently, we perform a non-targeted metabolomics analysis of the cells and find that cinobufagin exerts its anti-hepatoma effects by interfering with lipid metabolic reprogramming. The results show that cinobufagin mainly interferes with glycerophospholipid and fatty acyl levels. In addition, *in vivo* experiments reveal that cinobufagin restrains tumor growth without obvious adverse effects. Serum metabolomics analyses reveal that cinobufagin causes significant changes in the levels of lipid and lipid-like differential metabolites.

**Conclusion:**

Cinobufagin is a promising natural drug for the treatment of liver cancer, and PEXs may be potential molecular targets of cinobufagin to exert anticancer effects. Cinobufagin induces peroxisome dysfunction through suppression of the proteins encoded by the PEX genes and interferes with lipid metabolism to restrain the growth of liver cancer cells.

## 1 Introduction

Primary liver cancer is the sixth most commonly diagnosed cancer and the third most common cause of cancer death worldwide. According to the Globocan report, there were approximately 906,000 new cases and 830,000 deaths associated with primary liver cancer worldwide in 2020 (Sung et al., 2021). Notably, liver cancer is the leading cause of cancer deaths among men and women in Mongolia, Thailand, Cambodia, Egypt, and Guatemala. Primary liver cancers include hepatocellular carcinoma (HCC) and intrahepatic cholangiocarcinoma as well as other rare types, with HCC accounting for 75%–85% (Sung et al., 2021). Currently, the commonly used therapeutic methods for HCC include surgical resection, liver transplantation, chemotherapy, radiofrequency ablation, and targeted drugs (Llovet et al., 2018). Although surgical resection has consistently been considered an effective method for possible treatment of liver cancer, 70%–80% of patients are diagnosed at middle to advanced stages, losing the opportunity for surgical resection (Omata et al., 2017; Bruix and Llovet, 2002). Until now, chemotherapy has been the most common treatment option for advanced cancers. However, due to the serious side effects caused by chemotherapy drugs, the efficacy of chemotherapy drugs for liver cancer is not satisfactory (Ikeda et al., 2018). Sorafenib tosylate, durvalumab + tremelimumab, and durvalumab have better treatment outcomes among first-line liver cancer regimens in the 2023 NCCN Clinical Practice Guidelines in Oncology: Hepatocellular Carcinoma (Corbett et al., 2023). Therefore, the development of novel therapeutic drugs effective against liver cancer is critical.

The peroxisome is a subcellular organelle present in eukaryotic cells. Its main function is to participate in lipid metabolism such as the catabolism of very long chain fatty acids and the anabolism of plasmalogen (Jain et al., 2020). In addition, peroxisomes are also involved in hydrogen peroxide decomposition. The biogenesis of peroxisomes, primarily through both *de novo* synthesis and division, requires the involvement of peroxisomal proteins encoded by the PEX genes, also called peroxins. At present, more than 30 isoforms have been found, such as PEX2, PEX3, PEX5, PEX6, PEX10, and so on. Among the peroxins, PEX5 plays a transporter role, and PEX2, PEX10, and PEX12 are thought to form a complex that functions as an E3 ubiquitin ligase to help recycle PEX5 in a ubiquitin modification-dependent process (Brown et al., 2014). PEX gene mutations have been discovered in recent years to cause peroxisomal dysfunction, resulting in a range of diseases. Peroxisome defects may be associated with the pathogenesis of age-related neurodegenerative disorders, including Alzheimer’s disease and Parkinson’s disease, as well as age-related metabolic diseases, such as obesity, diabetes, and non-alcoholic fatty liver disease (Waterham et al., 2016). An accumulation of data suggests that peroxisomes play an active role in cancer. Recently, studies have shown that intervention with peroxins or the function of peroxisomes plays an active role in cancer treatment, a disease characterized by metabolic abnormalities (Cai et al., 2018; Benjamin et al., 2013). The survival of liver cancer cells may depend on peroxins (Cai et al., 2018). Given that peroxisomal proteins have been documented to promote tumorigenic events, inhibiting peroxisome function could represent a promising strategy for cancer therapy.

In recent years, many researchers have paid more attention to developing anticancer therapeutic strategies that focus on targeting cancer metabolism. Lipid metabolic reprogramming is one of the most prominent metabolic alterations in cancer. Lipids are generally classified as fatty acids, triacylglycerols, glycerophospholipids, sphingolipids, and steroids, which are necessary for building biofilms, storing energy, and transmitting signals. Key enzymes in lipid uptake, *de novo* synthesis, and catabolism in different types of cancer sustain accelerated tumor cell growth, invasion, and metastasis through abnormal expression or activation. Published studies have shown that key enzymes in the process of lipid metabolism, especially fatty acid synthetase (FASN) and ATP-citrate synthase (ACLY), are positively correlated with the occurrence and development of liver cancer; suppression of key enzymes in lipid metabolism could restrain the growth of liver cancer cells (Abdel-Magid, 2015; Lally et al., 2019). The newest oral FASN inhibitor (TVB2640) is currently in clinical trials for the treatment of liver cancer (Hu et al., 2020). Dysregulation of lipid metabolism plays an important role in the growth and metastasis of liver cancer. Intervention in abnormal lipid metabolism can be a candidate strategy for clinical liver cancer therapy. More importantly, published reports have revealed that peroxisomes participate in lipid metabolism, such as promotion of fatty acid oxidation and phospholipid synthesis, especially the ether phospholipid synthesis (Lodhi and Semenkovich, 2014). Enzymes involved in peroxisomal lipid processing are elevated in numerous tumor types. Research has shown that the regulation of tumor cell lipid metabolism by traditional Chinese medicine (TCM) intervention represents an effective approach for anticancer therapy (Wu et al., 2023; Wu et al., 2022).

Chansu is made by processing and drying the white serous fluid of the posterior ear and skin glands of *Bufo bufo gargarizans* Cantor or *Bufo melanostictus* Schneider. It has been used for nearly a thousand years, and it is usually used in combination with other traditional Chinese medicines to form formulas, such as Liushen pills, Baoxin pills, and toad poison pills. Chansu is widely used in China and other Asian countries to treat liver cancer and hepatitis B. The main bioactive chemical components of Chansu have been identified as bufadienolides and indole alkaloids. Bufadienolides have been proven to exhibit excellent antitumor activity. Cinobufagin, as a bufadienolide, has been reported as the main antitumor active component of Chansu. Many studies have shown that cinobufagin exerts antitumor effects by inhibiting cancer cell proliferation, promoting apoptosis, inducing cell cycle arrest, and enhancing the efficacy of other conventional chemotherapeutic agents (Yang et al., 2021; Ma et al., 2016). However, the effect of cinobufagin on peroxisomes in cancer cells has not been reported.

In this study, for the first time, we demonstrate that cinobufagin can cause peroxisomal dysfunction in HepG2 and SK-HEP-1 tumor cells and restrain the growth of tumor cells *via* suppression of peroxins PEX5 and PEX10. Further studies show that cinobufagin inhibits the expression of proteins associated with lipid metabolism through downregulation of peroxins. We identify that cinobufagin can lead to the reduction of several lipid levels (such as fatty acyls and glycerophospholipids). In addition, we demonstrate that cinobufagin restrains tumor growth of hepatoma H22-bearing Kunming mice *in vivo*. These results suggest that cinobufagin suppresses peroxisome function to interfere with lipid reprogramming, and, as a result, exerts anti-hepatoma effects. Our study provides a novel theoretical basis for the clinical application of Chansu and promotes the development of new drugs from traditional Chinese medicine.

## 2 Materials and methods

### 2.1 Drugs and antibodies

DMSO was purchased from Sigma. Cinobufagin (purity: 99.6%) was purchased from the National Institutes for Food and Drug Control (Beijing, China). Antibodies against FASN (ab128870) were obtained from Abcam (Cambridge, United Kingdom). Antibodies against PEX5 (sc-137103), PEX10 (sc-134419), PEX6 (sc-271813), catalase (sc-271803), and ABCD3 (sc-7351) were purchased from Santa Cruz Biotechnology (Santa Cruz, CA, United States). The 1% crystal violet stain solution was purchased from Solarbio Biotechnology (Beijing, China). Dulbecco’s modified Eagle medium (DMEM), fetal bovine serum (FBS), 0.25% trypsin-EDTA, and penicillin-streptomycin mixture were obtained from Corning Life Sciences (Steuben County, New York, United States).

### 2.2 Cell culture

HepG2 and SK-HEP-1 tumor cells were purchased from American Type Culture Collection (Manassas, VA, United States). Cells were cultured in DMEM containing 10% (FBS) fetal bovine serum and 1% streptomycin–penicillin. The cells were grown in an incubator containing a 5% CO_2_ humidified atmosphere at 37 °C.

### 2.3 Cell viability assay

In brief, cells were seeded into 96-well plates at a density of 3500 cells/well. The next day, the cells were exposed to fresh complete medium with various concentrations of cinobufagin for 24 h and 48 h, respectively. At the specified times, the supernatant solution was discarded, and 100 μL of 10% CCK-8 solution was added to each well. The plate was incubated for 2 h at 37 °C. Finally, the optical density of each sample at 450 nm was assayed using a microplate reader (Perkin-Elmer, Waltham, MA, United States).

### 2.4 Colony formation assay

Cells at the logarithmic phase were seeded in six-well plates with a density of 400 cells/well. After 24 h, the cells were treated with cinobufagin. The cell culture medium was changed every 3 days during the incubation. When clearly visible colonies appeared in the medium after 6–7 days of drug treatment, the supernatant was removed. After that, the cells were fixed with anhydrous methyl alcohol for 15 min and stained with 0.5% crystal violet solution for 15 min. Finally, the crystal violet solution was discarded, and phosphate-buffered saline was used to wash the cells three times. A digital camera was used to take pictures of the cell colonies (Lecia Microsystems GmbH, Wetzlar, Germany).

### 2.5 Immunofluorescence assay

HepG2 and SK-HEP-1 cells were seeded in 1.5 cm confocal dishes. After 1 day, the cells were treated with cinobufagin (0 and 500 nM) for 24 h. Then, the cells were gently washed three times with phosphate buffered saline (PBS) and fixed in 4% paraformaldehyde for 10 min. Next, the cells were permeabilized with PBS containing 0.5% Triton X-100 for 20 min at room temperature. Then the cells were blocked with 1% BSA solution at 37 °C for 1 h. The blocking solution was removed, and the samples were incubated overnight at 4 °C with the primary antibodies against ABCD3 (1:200). The next day, the samples were washed three times with PBS for 5 min each time. The samples were then incubated with Alexa Fluor 488-conjugated anti-mouse secondary antibody (1:200) for 1 h at 37 °C. Next, the nuclei were counterstained with 10 μg/mL DAPI at room temperature. Finally, the samples were washed with PBS, and cells were observed and photographed under an Olympus FV1000 confocal laser scanning microscope (Olympus, Tokyo, Japan).

### 2.6 Western blotting

HepG2 and SK-HEP-1 cells were seeded in 6-well plates. On the second day, cells were treated with different concentrations of cinobufagin (0, 31.25 nM, 62.5 nM, and 125 nM) for 24 h. The cells were washed with 1 mL PBS and lysed in cell lysis buffer. Next, the lysates were heated in a metal bath at 95 °C for 5 min. The protein samples were then separated on SDS-PAGE gel and transferred to polyvinylidene fluoride (PVDF) membranes. The PVDF membranes were blocked overnight in Tris-buffered saline with Tween 20 (TBST) solution containing 5% nonfat milk powder. Next, the membranes were blocked and probed with primary antibodies against catalase, ABCD3, ACLY, PEX5, PEX6, PEX10, FASN, and *β*-actin (1:1000) at 4 °C overnight. Subsequently, the membranes were washed with TBST for 40 min and incubated with secondary antibodies for 2 h at room temperature. Finally, PVDF membranes were washed with TBST for 40 min and then incubated using an enhanced chemiluminescence (ECL) detection reagent and gel imaging system (Azure Biosystems C600, Dublin, CA, United States) to visualize proteins.

### 2.7 Cell transfection

HepG2 and SK-HEP-1 cells were seeded in six-well plates. When the cell confluence reached 60%–70%, the medium was replaced with Opti-MEM for 6 h. Then, 5 μL of the Lipofectamine reagent and 10 μL of siRNA (20 μM) were added to 2 mL of Opti-MEM, mixed, and left to stand for 20 min. The cells were incubated with the above solution instead of the medium for 6 h. Then the medium was replaced with normal complete medium to culture the cells.

siRNA sequences were as follows: PEX5: 5′-CGUAUCCUGGGAUCUCUCU-3′; PEX6: 5′-GUGUGAUGGCACUUAGAACGCGUAGCU-3′; PEX10: 5′-GGUUGAGCUGCUCUCAGAU-3′; Negative Control (NC): 5′-UUCUCCGAACGUGUCACGUTT -3′.

### 2.8 Untargeted metabolomics analysis

A total of 8 × 10^6^ HepG2 cells were seeded in 10 cm dishes. On the second day, cells were treated with different concentrations of cinobufagin (0 and 1 μM) for 24 h. The cells were washed with PBS, resuspended in methanol, and crushed using an ultrasonic crusher. Next, the supernatant was collected, freeze-dried, and dissolved in 10% methanol for UHPLC–MS/MS analysis conducted by Novogene Co., Ltd. (Beijing, China) as previously described (Yang et al., 2023). Principal component analysis (PCA) and partial least squares discriminant analysis (PLS-DA) were performed using the metabolomics data processing software MetaX. The statistical significance (*p*-value) of each metabolite was calculated using univariate analysis (*t*-tests), and the fold change (FC) between the two groups was determined. The screening of differentially expressed metabolites was primarily based on three parameters: VIP, FC, and *p*-value. Differentially expressed metabolites were identified by setting thresholds of VIP >1.0, FC ≥ 2 or FC ≤ 0.5, and *p*-value <0.05.

Metabolite extraction from serum samples, UHPLC–MS/MS analysis, and data processing were all performed by Novogene Co., Ltd. (Beijing, China). Data processing was carried out using Compound Discoverer 3.3, and metabolite identification was achieved by matching against the mzCloud, mzVault, and MassList databases. A total of eight serum samples were included in this study, with four samples in the control group and four in the treatment group. Each group consisted of four independent biological replicates. Multivariate statistical analysis was performed using metaX software. Principal component analysis (PCA) was used to examine the separation trend between groups, and the results showed a clear separation between the two groups. Supervised partial least squares discriminant analysis (PLS-DA) was then conducted to establish a discriminant model, and the model parameters (R^2^Y, Q^2^Y) were calculated using 7-fold cross-validation. Metabolites that simultaneously met the following criteria were defined as differentially expressed metabolites: VIP >1.0 (obtained from the PLS-DA model), *p* < 0.05 (two-tailed t-test), and FC > 1.2 or FC < 0.833. Volcano plots were generated to visualize the overall distribution of differential metabolites. The metabolite data were normalized by z-scores before generating clustering heatmaps using the R package pheatmap.

### 2.9 Transcriptome sequencing

Total RNA was extracted from HepG2 cells, treated with or without cinobufagin (1 μM) for 24 h, using the E.Z.N.A.® Total RNA Kit I. The statistical criteria for selecting differentially expressed genes (DEGs) were defined using a |log_2_FC|≥1 and FDR ≤0.001. The RNA samples were then subjected to sequencing on the BGISEQ-500 platform by Beijing Genomics Institute (BGI, Shenzhen, China).

### 2.10 H22 hepatoma-bearing mice model

Male Kunming mice (4–5 weeks old, 18–20 g) were purchased from Jinan Pengyue Experimental Animal Breeding Co., Ltd. The tumor cell suspension of murine H22 hepatoma cells was prepared with PBS at a concentration of 8.0 × 10^6^ cells/mL. The mice were inoculated in the right posterior dorsal region with the tumor cell suspension (200 µL for each mouse) to establish the tumor-bearing mouse model. The tumor-bearing mice were divided into the control group, the positive treatment group, and the cinobufagin treatment groups, which were divided into high- and low-dose groups. Ten mice were used in each cohort. Twenty-four hours later, the positive control group was intraperitoneally injected with 5-fluorouracil (5-FU, 30 mg/kg, once every other day). The control group mice received the same volume of PBS intraperitoneally. The mice in the cinobufagin treatment groups were treated intraperitoneally with 1 mg/kg for the low dose group (LD) and 2 mg/kg for the high dose group (HD) per day, respectively (cinobufagin was dissolved in PBS). All groups of mice were treated for 13 days. All mice were sacrificed by cervical dislocation, and tumors were dissected on the 13th day. Tumor volume (mm^3^) was calculated by the formula: length × width × (width/2). Tumor size was measured and normalized to the initial volumes, and body weights of mice were also recorded. The animal experiment was performed in accordance with the guidelines for the use and care of animals approved by the Ethics Committee of Binzhou Medical University (Animal Ethics Approval Number: 2023-409).

### 2.11 Blood analysis

Blood samples were collected from Kunming mice. The samples were collected for serum biochemical analysis of alanine transaminase (ALT) and aspartate transaminase (AST).

### 2.12 Hematoxylin and eosin staining

Briefly, tumor tissues or major organs such as the heart, liver, spleen, lung, and kidney of Kunming mice were fixed in 4% paraformaldehyde and then embedded in paraffin. Subsequently, the paraffin sections were subjected to H&E staining.

### 2.13 Statistical analysis

Using the software GraphPad Prism 5.0, the two-tailed Student’s t-test or two-way analysis of variance was used to determine the significance of the difference between the groups. Data are expressed as mean ± SEM. *P* < 0.05 was considered to be statistically significant.

## 3 Results

### 3.1 Cinobufagin inhibits the expression of ABCD3 and catalase

The chemical structure of cinobufagin is shown in Figure 1A. In our previous study, we demonstrated that cinobufagin could restrain growth, trigger cell cycle arrest, promote reactive oxygen species (ROS) production, and induce disruption of the mitochondrial membrane potential of HepG2 and SK-HEP-1 cells, with an IC50 of 95 nM to HepG2 cells. Transcriptome sequencing was used to investigate the underlying mechanisms of antitumor effects of cinobufagin (Yang et al., 2023; Yang et al., 2021). The KEGG enrichment analysis of the differentially expressed genes identified by transcriptome sequencing revealed that the intervention of peroxisomes was partially responsible for the antitumor effects of cinobufagin. Peroxisomes are known to house enzymes involved in many biochemical reactions, of which catalase is the hallmark enzyme of peroxisomes, accounting for about 40% of the total peroxisome enzymes (Nagotu et al., 2012). Catalase is present in various animal tissues and is particularly highly expressed in the liver. Another commonly used marker of peroxisomes is the ABCD transporter family member ABCD3 (also known as PMP70), a 70 kDa peroxisome membrane protein. ABCD3 is a major component of the peroxisome membrane and is involved in the transport of lipid derivatives through the peroxisome plasma membrane (Dahabieh et al., 2018). ABCD3 is frequently used as a marker of peroxisome number, and catalase is also used as a marker of peroxisomal function.

**FIGURE 1:**
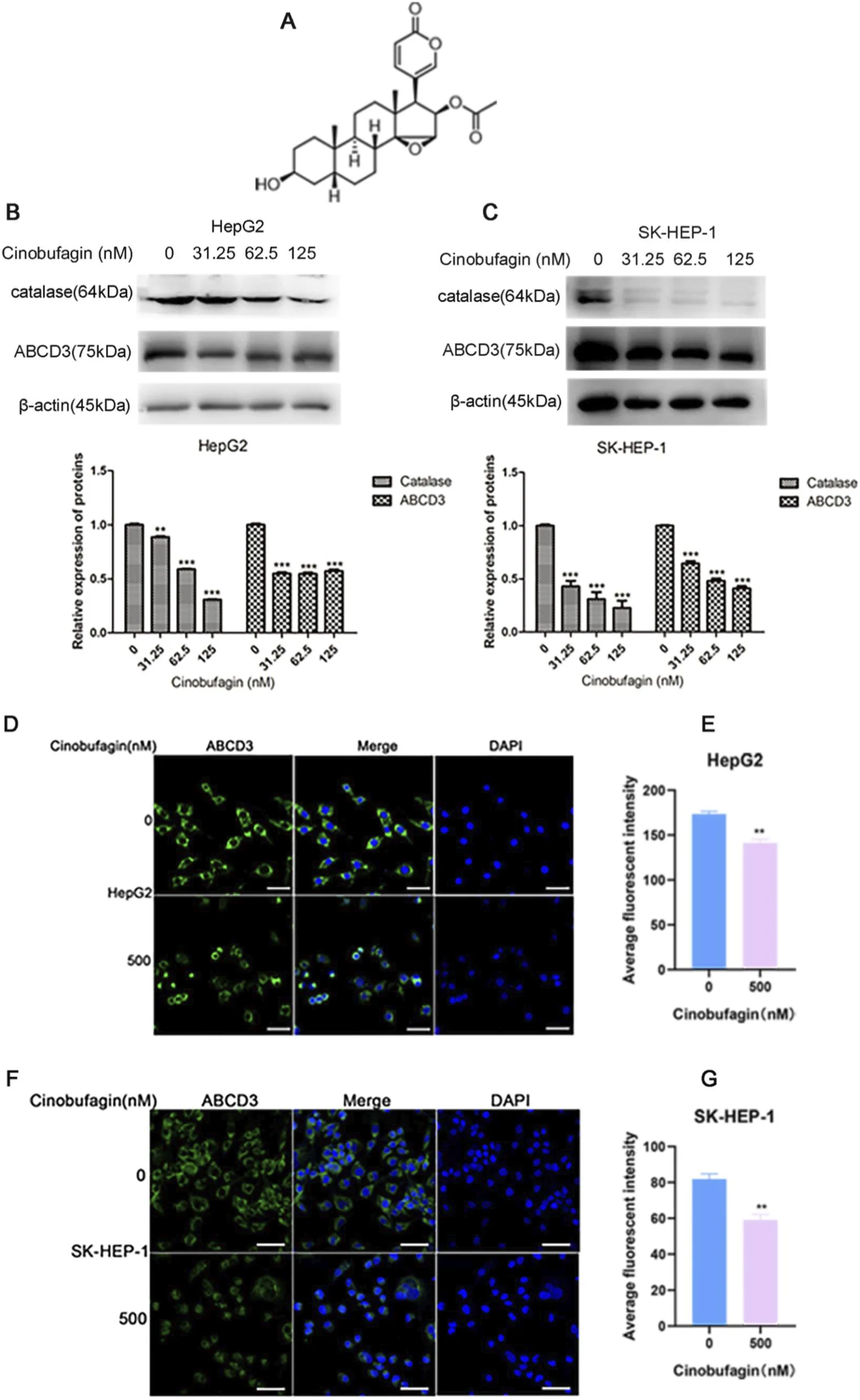
Cinobufagin induces peroxisome dysfunction in HepG2 and SK-HEP-1 tumor cells. **(A)** Chemical structure of cinobufagin. **(B,C)** HepG2 and SK-HEP-1 cells were treated with cinobufagin at the concentrations of 0, 31.25 nM, 62.5 nM, and 125 nM for 24 h. Western blotting analysis was carried out to detect changes in the protein levels of catalase and ABCD3 in HepG2 and SK-HEP-1 tumor cells. **(D,F)** HepG2 and SK-HEP-1 cells treated with or without cinobufagin (500 nM) for 24 h were fixed and incubated with anti-ABCD3 antibody. The nuclei (blue) and ABCD3 foci (green) were visualized with laser confocal microscopy. **(E,G)** Fluorescence intensity analysis. Representative images are presented. Scale bar, 20 μm. ^**^ *p* < 0.01, ^***^ *p* < 0.001. The data are representative of three independent experiments, and each was performed at least in triplicate.

To investigate the effect of cinobufagin on these two representative peroxisome markers, we analyzed the expression of ABCD3 and catalase in HepG2 and SK-HEP-1 tumor cells by Western blotting. The results showed that the expression of ABCD3 and catalase in HepG2 and SK-HEP-1 cells was significantly reduced in response to cinobufagin (Figures 1B,C). We also detected ABCD3 by immunofluorescence assay. As shown in Figures 1D–G, a significant reduction of average fluorescence intensity was observed in HepG2 and SK-HEP-1 tumor cells exposed to cinobufagin, indicating a decrease in the number of peroxisomes. These results reveal that cinobufagin impairs peroxisomal function in HepG2 and SK-HEP-1 tumor cells. In addition, peroxisomes are involved in the removal of ROS, of which catalase is the major force in removing ROS. Our previous study revealed that cinobufagin induced ROS production, and suppression of peroxisomal function may be partially responsible for the ROS production of tumor cells in response to cinobufagin (Yang et al., 2023).

### 3.2 Cinobufagin inhibits the expression of PEX5, PEX6, and PEX10 in HepG2 and SK-HEP-1 tumor cells

A variety of proteins on the peroxisome membrane are called peroxisome membrane proteins (PMP). Among them, a class of proteins that play an essential role in peroxisome biogenesis is called peroxisome proteins. They are encoded by the PEX gene. PEX5 acts as a transporter protein. Moreover, PEX2, PEX10, and PEX12 are thought to form a complex that acts as an E3 ubiquitin ligase to help recycle PEX5 *via* ubiquitin modification-dependent processes (Chen et al., 2002; Brown et al., 2014). After the receptor protein releases the matrix protein into the peroxisome, monoubiquitinated PEX5 is recognized by a hexamer AAA ATPase, which consists of PEX1 and PEX6. Deletion of the PEX5 gene leads to peroxisome biogenesis defects and peroxisome dysfunction. Studies have shown that peroxisome proteins have a certain relationship with the occurrence of cancer, and targeting peroxisome proteins may be a potential strategy for cancer treatment (Wen et al., 2026). In this study, based on transcriptome data analysis, we found that multiple PEX genes were downregulated in the cinobufagin-treated group compared with the control group (Table 1). Subsequently, we examined the expression of PEX5, PEX6, and PEX10 in response to cinobufagin in HepG2 and SK-HEP-1 tumor cells. Western blotting results showed that the expression of PEX5, PEX6, and PEX10 was decreased after treatment with cinobufagin (Figures 2A,B).

**TABLE 1:** Details of the differentially expressed genes identified in cancer cells treated with or without cinobufagin based on transcriptomic data**.**

Gene_symbol	Up/down	log_2_ fc_HepG2_CA-vs-HepG2_HA
PEX5	Down	−1.83
PEX6	Down	−3.36
PEX10	Down	−2.75
PEX1	Down	−6.67
PEX2	Down	−1.31
PEX7	Down	−2.67
PEX11B	Down	−3.83
PEX11G	Down	−3.65
PEX12	Down	−4.77
PEX14	Down	−1.20
FASN	Down	−2.87
ACLY	Down	−2.87
ECH1	Down	−1.25
ABCD3	Down	−1.31
CAT	Down	−4.13

CA, control group, HA. cinobufagin treatment group, fc, fold change.

**FIGURE 2:**
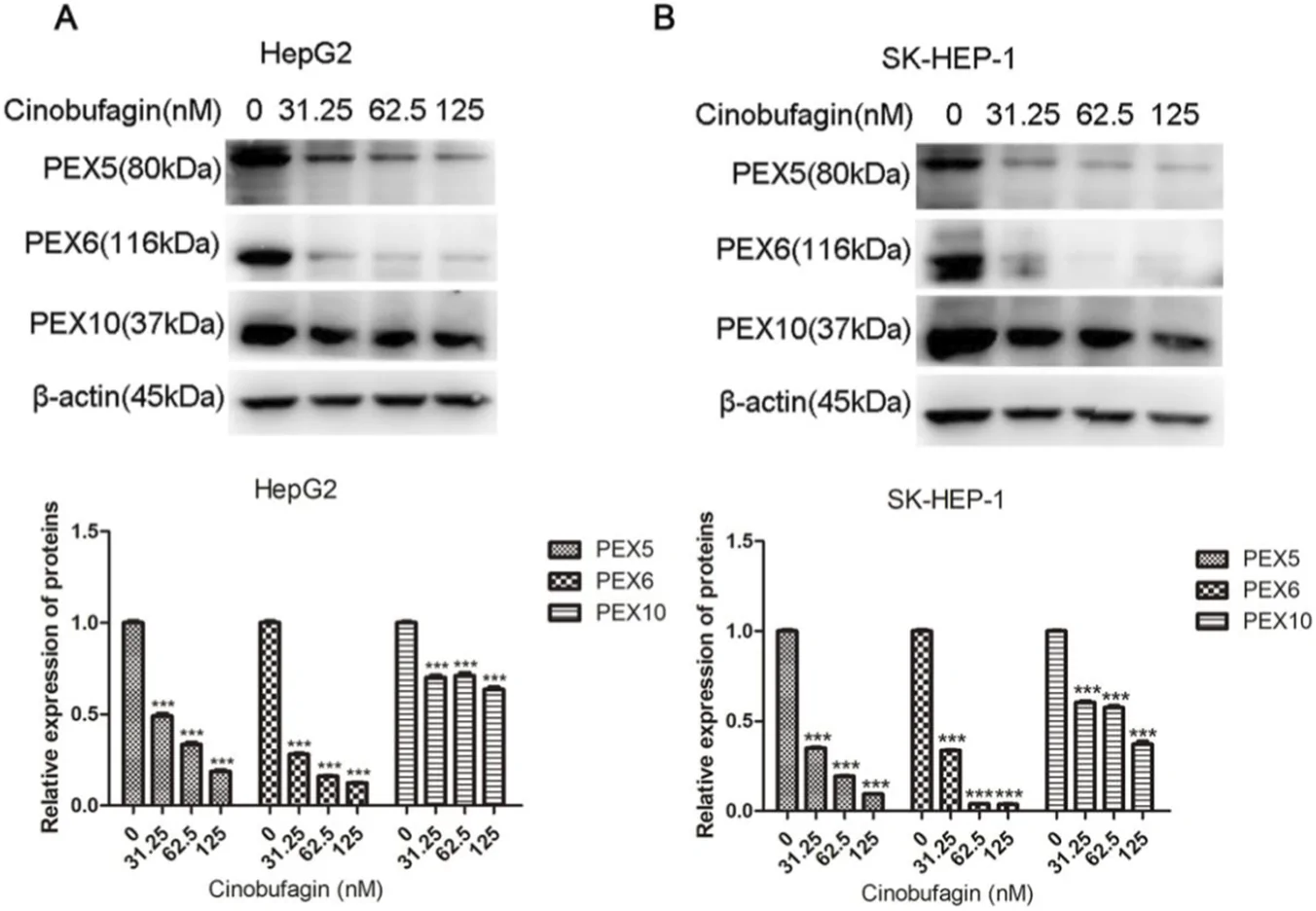
Cinobufagin inhibits the expression of PEX5, PEX6, and PEX10 proteins in HepG2 and SK-HEP-1 tumor cells. The expression of PEX5, PEX6, and PEX10 in HepG2 **(A)** and SK-HEP-1 **(B)** cells incubated with cinobufagin at different concentrations for 24 h was detected by Western blotting. ^***^ *p* < 0.001. The data are representative of three independent experiments, and each was performed at least in triplicate.

### 3.3 Cinobufagin inhibits the growth of HepG2 and SK-HEP-1 tumor cells by downregulating PEX5 and PEX10

To examine the effects of PEX5, PEX6, and PEX10 on the growth of HepG2 and SK-HEP-1 tumor cells, we silenced PEX5, PEX6, or PEX10 in HepG2 and SK-HEP-1 cells by RNA interference. As shown in Figures 3A–D, knockdown of PEX5 significantly suppressed the proliferative potential and colony formation of cancer cells. Additionally, depletion of PEX5 enhanced the blocking effects of cinobufagin on the proliferation of HepG2 and SK-HEP-1 cells. As shown in Figures 4A–D, PEX10 knockdown inhibited the proliferation and colony formation of cancer cells. In addition, depletion of PEX10 enhanced the blocking effects of cinobufagin on the proliferation of tumor cells. However, as shown in Figures 5A,B, knockdown of PEX6 had no effect on the proliferation of HepG2 and SK-HEP-1 tumor cells. This discrepancy may be related to functional specificity among members of the PEX protein family or cell type specificity.

**FIGURE 3:**
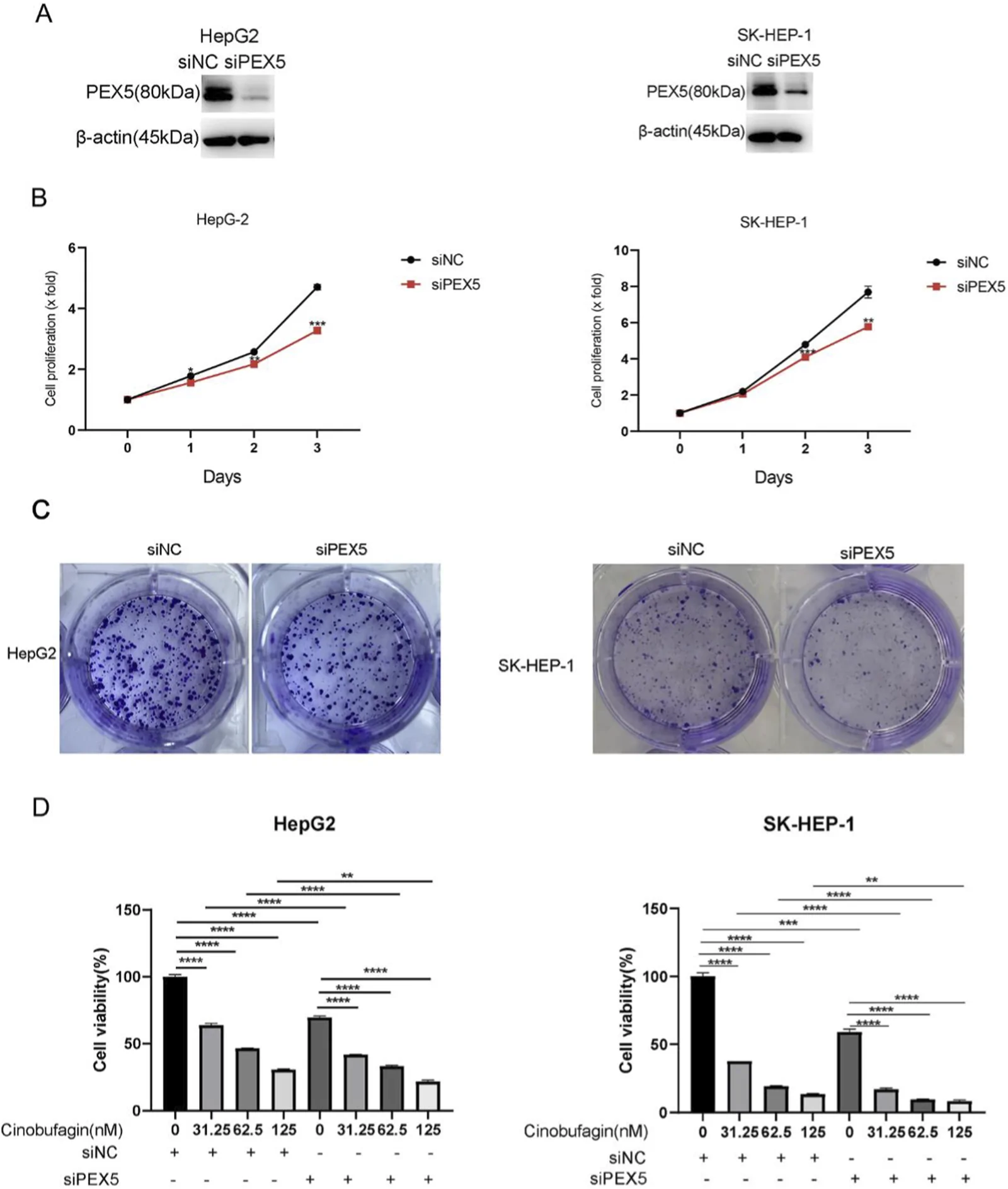
Cinobufagin inhibits the growth of HepG2 and SK-HEP-1 tumor cells by downregulating PEX5. **(A)** PEX5 was knocked down in HepG2 and SK-HEP-1 cells, and the PEX5 protein levels were detected by Western blotting. **(B)** HepG2 and SK-HEP-1 cells transfected with PEX5 siRNAs or negative control (NC) siRNAs were subjected to a cell proliferation assay. **(C)** HepG2 and SK-HEP-1 cells transfected with PEX5 siRNAs or NC siRNAs were subjected to a colony formation assay. **(D)** HepG2 and SK-HEP-1 cells transfected with PEX5 siRNAs or NC siRNAs were treated with or without cinobufagin at the concentrations of 0, 31.25 nM, 62.5 nM, and 125 nM for 48 h, and cell viability was measured using the CCK-8 assay. ^*^ *p* < 0.05, ^**^ *p* < 0.01, ^***^ *p* < 0.001, and ^****^ *p* < 0.0001. The data are representative of three independent experiments, and each was performed at least in triplicate.

**FIGURE 4:**
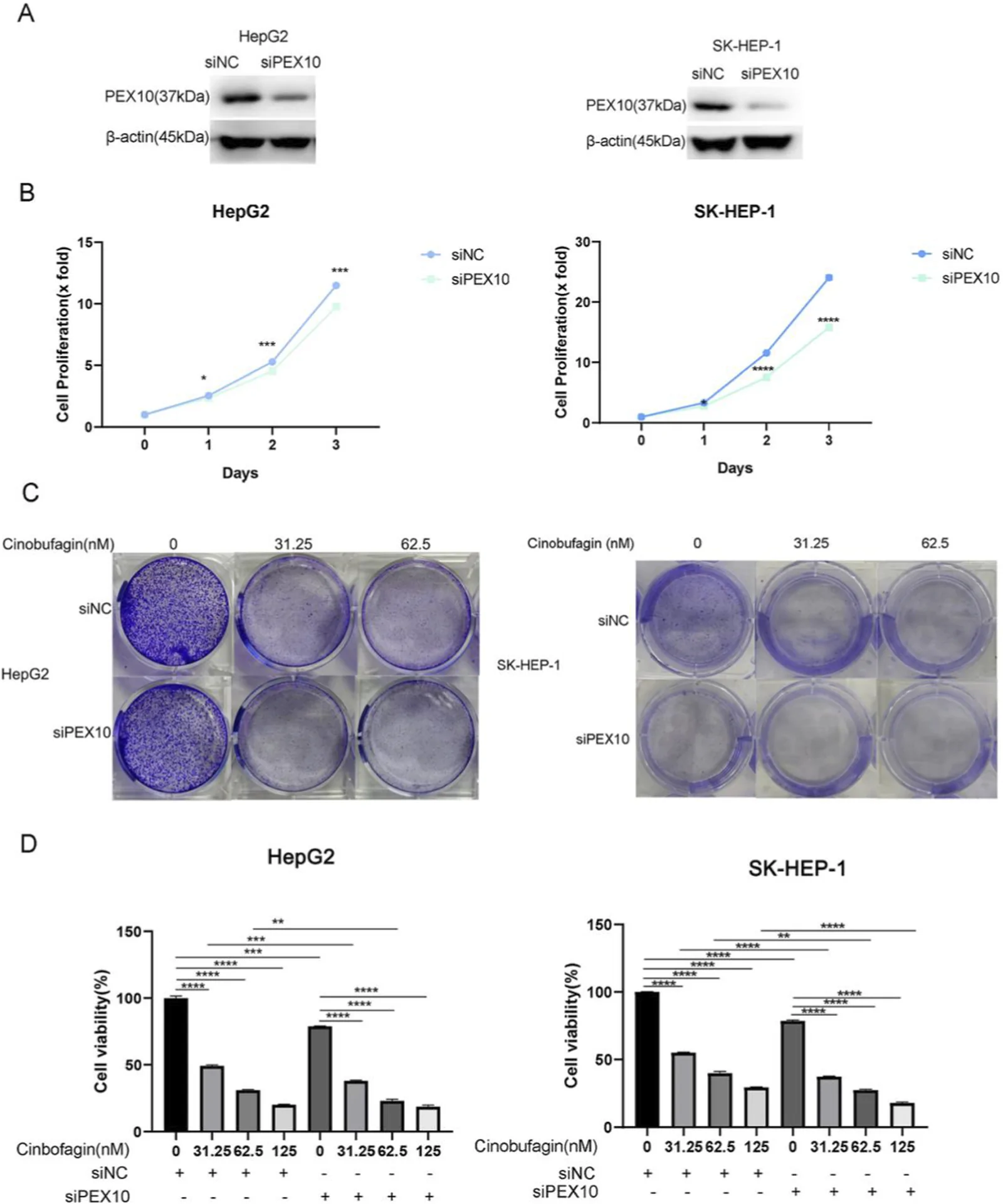
Cinobufagin inhibits the growth of HepG2 and SK-HEP-1 tumor cells by downregulating PEX10. **(A)** PEX10 was knocked down in HepG2 and SK-HEP-1 cells, and the PEX10 protein level was detected by Western blotting. **(B)** HepG2 and SK-HEP-1 cells transfected with PEX10 siRNAs or negative control (NC) siRNAs were subjected to a cell proliferation assay. **(C)** HepG2 and SK-HEP-1 cells transfected with PEX10 siRNA or NC siRNA were treated with cinobufagin at concentrations of 0, 31.25 nM, 62.5 nM, and 125 nM for 48 h, respectively, for a colony formation assay. **(D)** HepG2 and SK-HEP-1 cells transfected with PEX10 siRNAs or NC siRNAs were treated with or without cinobufagin at concentrations of 0, 31.25 nM, 62.5 nm, and 125 nM for 48 h. Cell viability was measured by CCK-8 assay. ^*^ *p* < 0.05, ^**^ *p* < 0.01, ^***^ *p* < 0.001, ^****^ *p* < 0.0001. The data are representative of three independent experiments, and each was performed at least in triplicate.

**FIGURE 5:**
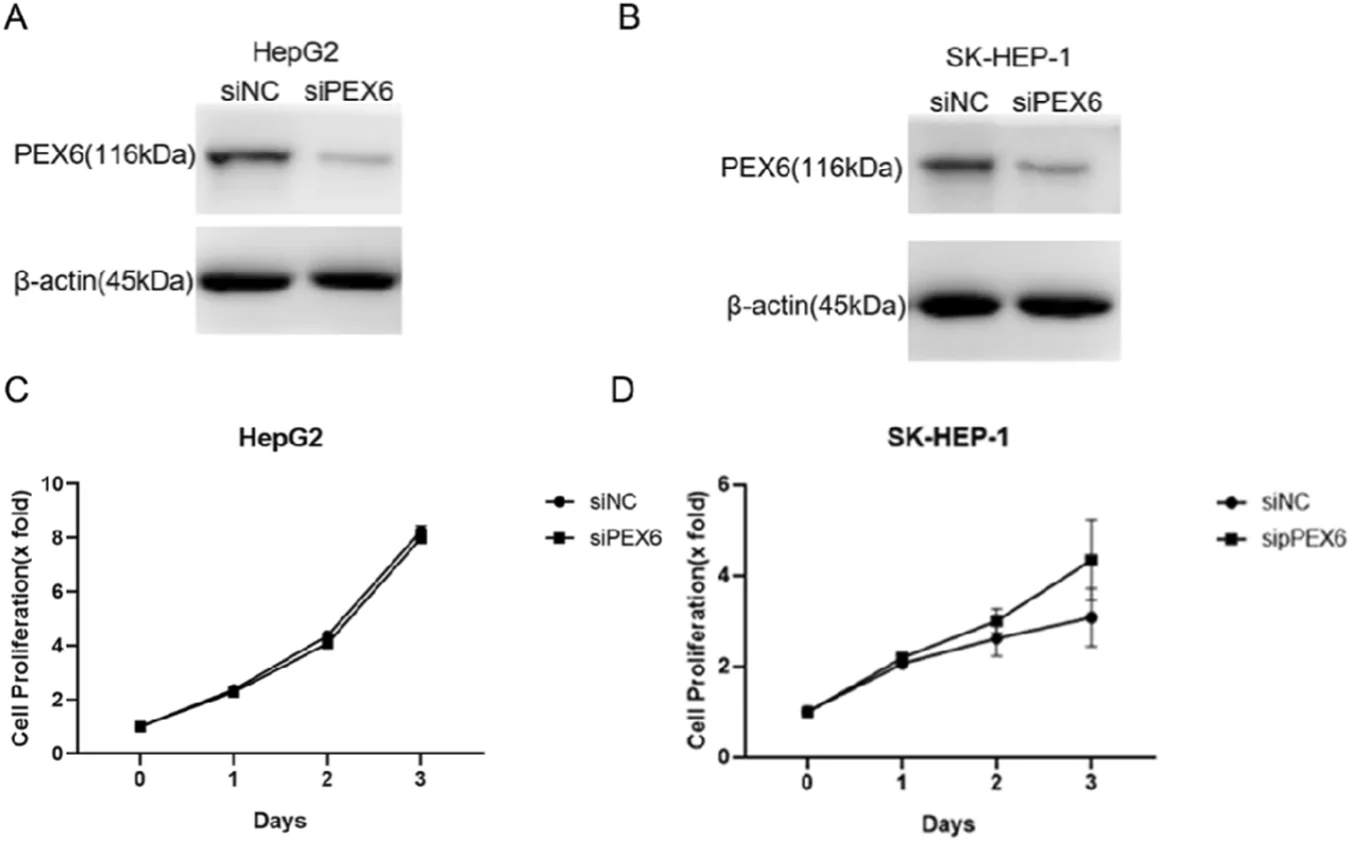
PEX6 knockdown does not affect the proliferation of HepG2 and SK-HEP-1 tumor cells. PEX6 was knocked down in **(A)** HepG2 and **(B)** SK-HEP-1 cells. The PEX6 protein level was detected by Western blotting. **(C,D)** HepG2 and SK-HEP-1 cells transfected with PEX6 siRNAs or negative control (NC) siRNAs were subjected to a cell proliferation assay. The data are representative of three independent experiments, and each was performed at least in triplicate.

### 3.4 Cinobufagin suppresses FASN, ACLY, and ECH1 expression in HepG2 and SK-HEP-1 tumor cells

Metabolic reprogramming plays an important role in tumor development, and increased synthesis of novel fatty acids is an essential metabolic feature of cancer. It has been shown that abnormal lipid metabolism occurs in a variety of cancers, including liver cancer. It is characterized by accelerated lipid uptake and biosynthesis (Fhaner et al., 2012; Wong et al., 2025). Abnormally accelerated fatty acid synthesis is required for tumor development. Studies have confirmed that genes encoding key enzymes of fatty acid synthesis are overexpressed in most cancer cells, such as FASN and ACLY, which induce the conversion of citric acid into fatty acid and acetyl-CoA, respectively. Overexpression of FASN is positively correlated with malignant proliferation and poor prognosis (Fan et al., 2024). It has been shown that suppression of FASN appears to be a good strategy for cancer treatment (Nuzzo et al., 2025). ACLY functions as a crucial catalytic enzyme in lipid metabolism and is crucial for fast-growing tumors. Published reports have shown that ACLY is highly expressed in tumor tissues, such as lung cancer, prostate cancer, and liver cancer (Fan et al., 2024). Inhibition of ACLY has been confirmed to be a potential target for cancer therapy (Liang et al., 2024). Enoyl coenzyme A hydratase (ECH1), which is a key enzyme in fatty acid *β*-oxidation, exists in peroxisomes and mitochondria. The liver is the main site of fatty acid catabolism. Some recent studies have shown that abnormal expression of ECH1 has a certain relationship with liver cancer (Zhang et al., 2025, 2013). We investigated the effects of cinobufagin on key enzymes in lipid metabolism. The results showed that cinobufagin restrained the expression of FASN, ACLY, and ECH1 in HepG2 and SK-HEP-1 tumor cells (Figures 6A,B).

**FIGURE 6:**
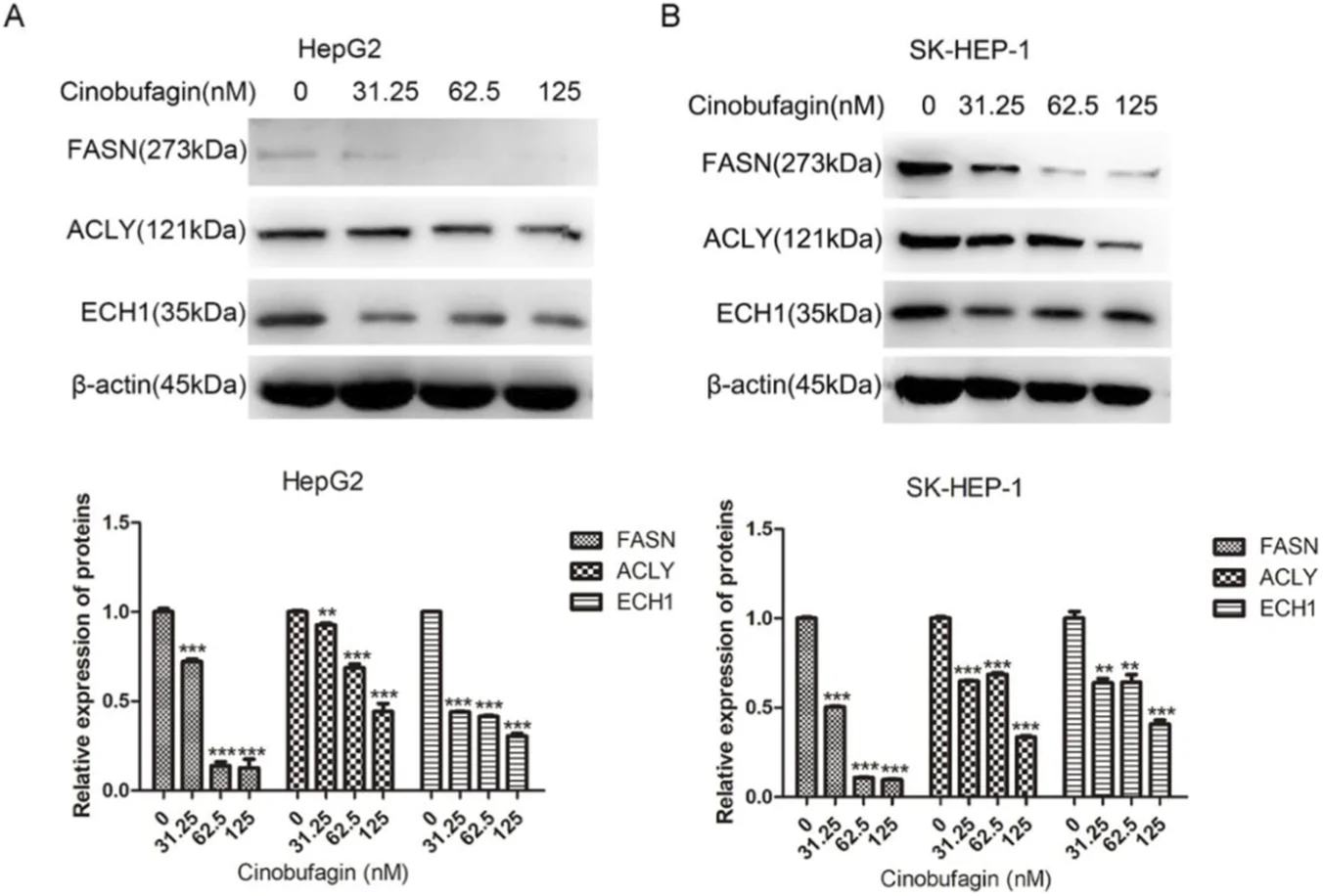
Cinobufagin suppresses FASN, ACLY, and ECH1 expression in HepG2 and SK-HEP-1 tumor cells. The expression of FASN, ACLY, and ECH1 in HepG2 **(A)** and SK-HEP-1 **(B)** cells incubated with cinobufagin at different concentrations for 24 h was detected by Western blotting. ^**^ *p* < 0.01, ^***^ *p* < 0.001. The data are representative of three independent experiments, and each was performed at least in triplicate.

### 3.5 PEX5 or PEX10 knockdown restrains the expression of enzymes related to lipid metabolism in HepG2 and SK-HEP-1 tumor cells

Peroxisomes participate in lipid metabolism, such as promotion of fatty acid oxidation and phospholipid synthesis, especially ether phospholipid synthesis. Peroxisomes contain many different proteins, which include peroxins, ABCD transporters, and several matrix enzymes. Published studies have identified interactions of ABCD1 with proteins involved in lipid metabolism, such as FASN and ACLY (Lally et al., 2019; Abdel-Magid, 2015). In our study, for the first time, we investigated the effects of PEX5 or PEX10 deletion on key enzymes involved in lipid metabolism in HepG2 and SK-HEP-1 tumor cells. As shown in Figure 7A, PEX5 knockdown inhibited the expression of FASN and ACLY in HepG2 and SK-HEP-1 tumor cells. Additionally, depletion of PEX5 had no effect on the expression of ECH1 in HepG2 and SK-HEP-1 cells. As shown in Figure 7B, knockdown of PEX10 suppressed FASN expression in HepG2 and SK-HEP-1 tumor cells and had no effect on the expression of ACLY and ECH1. These results suggest that cinobufagin restrains key enzymes in lipid metabolism partially through suppression of peroxisome proteins PEX5 and PEX10 in HepG2 and SK-HEP-1 tumor cells.

**FIGURE 7:**
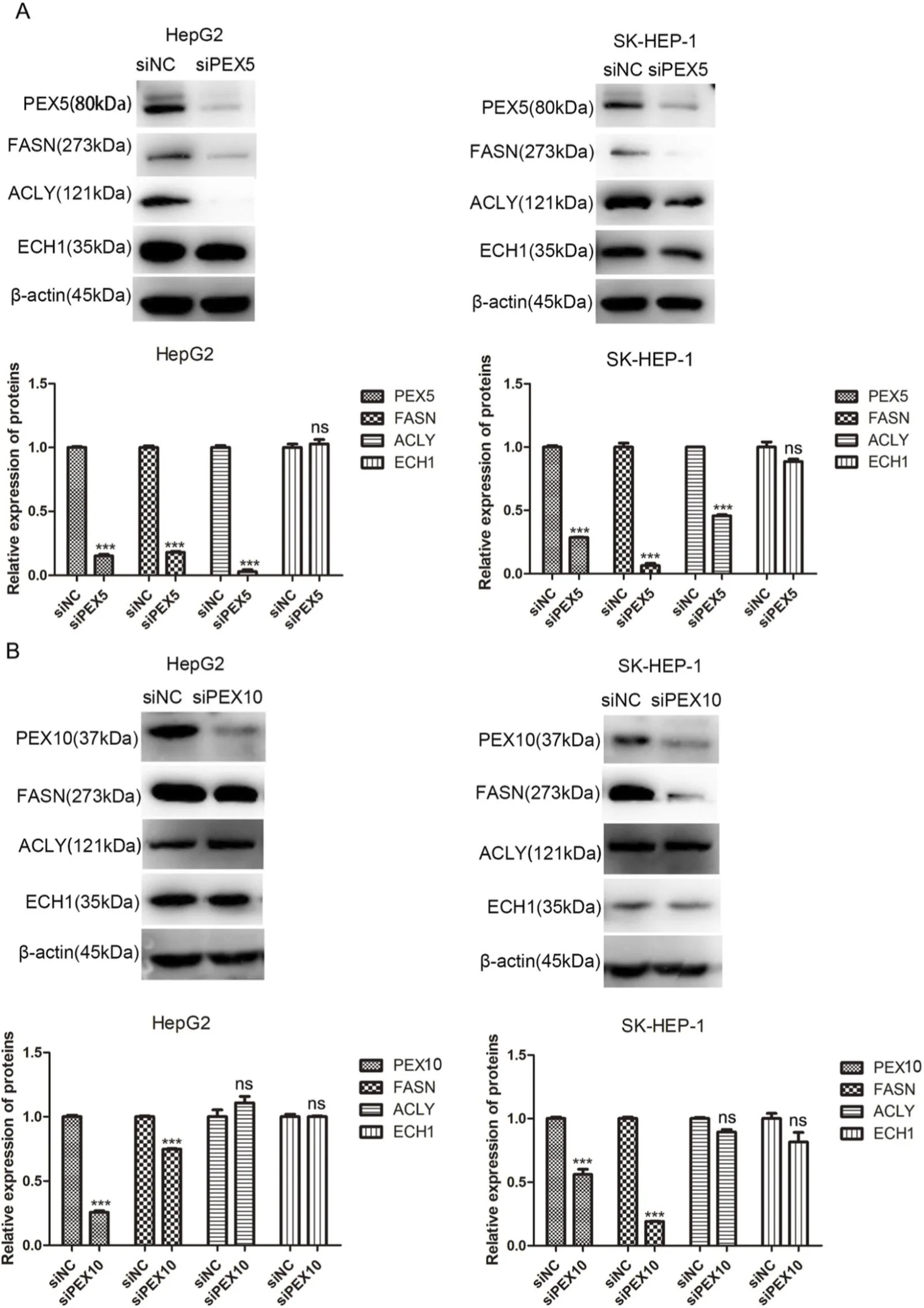
PEX5 or PEX10 knockdown restrains the expression of enzymes related to lipid metabolism in HepG2 and SK-HEP-1 tumor cells. **(A)** PEX5 was knocked down in HepG2 and SK-HEP-1 cells. FASN, ACLY, and ECH1 protein levels were detected by Western blotting. **(B)** PEX10 was knocked down in HepG2 and SK-HEP-1 cells. FASN, ACLY, and ECH1 protein levels were detected by Western blotting. ^***^ *p* < 0.001. The data are representative of three independent experiments, and each was performed at least in triplicate.

### 3.6 Cinobufagin suppresses the levels of fatty acyls and glycerophospholipids in liver cancer cells

Based on results indicating that cinobufagin restrains the key enzymes in lipid metabolism, we focused on the lipid-related metabolites in response to cinobufagin in human hepatoma cells *via* metabolomics technology. Figure 8A showed the chemical classification and proportion of lipid metabolites in HepG2 cells in response to cinobufagin. These lipid metabolites were divided into four categories (fatty acyls [FA], glycerophospholipids [GP], sphingolipids [SP], and others), accounting for 38%, 44%, 17%, and 1%, respectively. Among them, GP occupied the largest proportion, and lysophosphatidyl choline, lysophosphatidyl ethanolamine, and lysophosphatidyl serine accounted for 39%, 37%, and 13%, respectively (Figure 8B). Cinobufagin interfered with glycerophospholipid and fatty acyl levels, in detail, cinobufagin reduced the levels of fatty acyls (10-undecenoic acid, mevalonic acid, cis-2-decenoic acid, 16-hydroxyhexadecanoic acid, lipoamide, elaidic acid, propionylcarnitine, hexanoylcarnitine, acetyl-L-carnitine, 10-nitrolinoleate, 2-methylbutyroylcarnitine, and methyl palmitate) (Figure 8C). Additionally, as shown in Figure 8D, the levels of glycerophospholipids such as lysophosphatidyl ethanolamine (LPE), including LPE (19:1), LPE (20:1), LPE (20:2), LPE (20:3), LPE (20:4), LPE (20:5), LPE (14:0), LPE (16:1), LPE (17:0), LPE (17:1), LPE (18:0), LPE (18:1), LPE (18:2); lysophosphatidyl choline (LPC), including LPC (22:4), LPC (20:2), LPC (18:1), LPC (18:0), LPC (14:0), LPC (16:0), LPC (17:1), LPC (20:3), LPC (20:4), LPC (14:1), LPC (17:0), LPC (20:5), LPC (16:1), LPC (19:1); lysophosphatidyl serine (LPS), LPS (16:1), LPS (17:1), LPS (18:2), LPS (20:3), LPS(22:6); and phosphatidyl choline (PC) PC (9:0/9:0) were suppressed in liver cancer cells exposed to cinobufagin. Other glycerophospholipids, including 1-palmitoyl-sn-glycero-3-phosphocholine, 1-oleoyl-sn-glycero-3-phosphocholine, and choline glycerophosphate, were also reduced in response to cinobufagin (Figure 8E).

**FIGURE 8:**
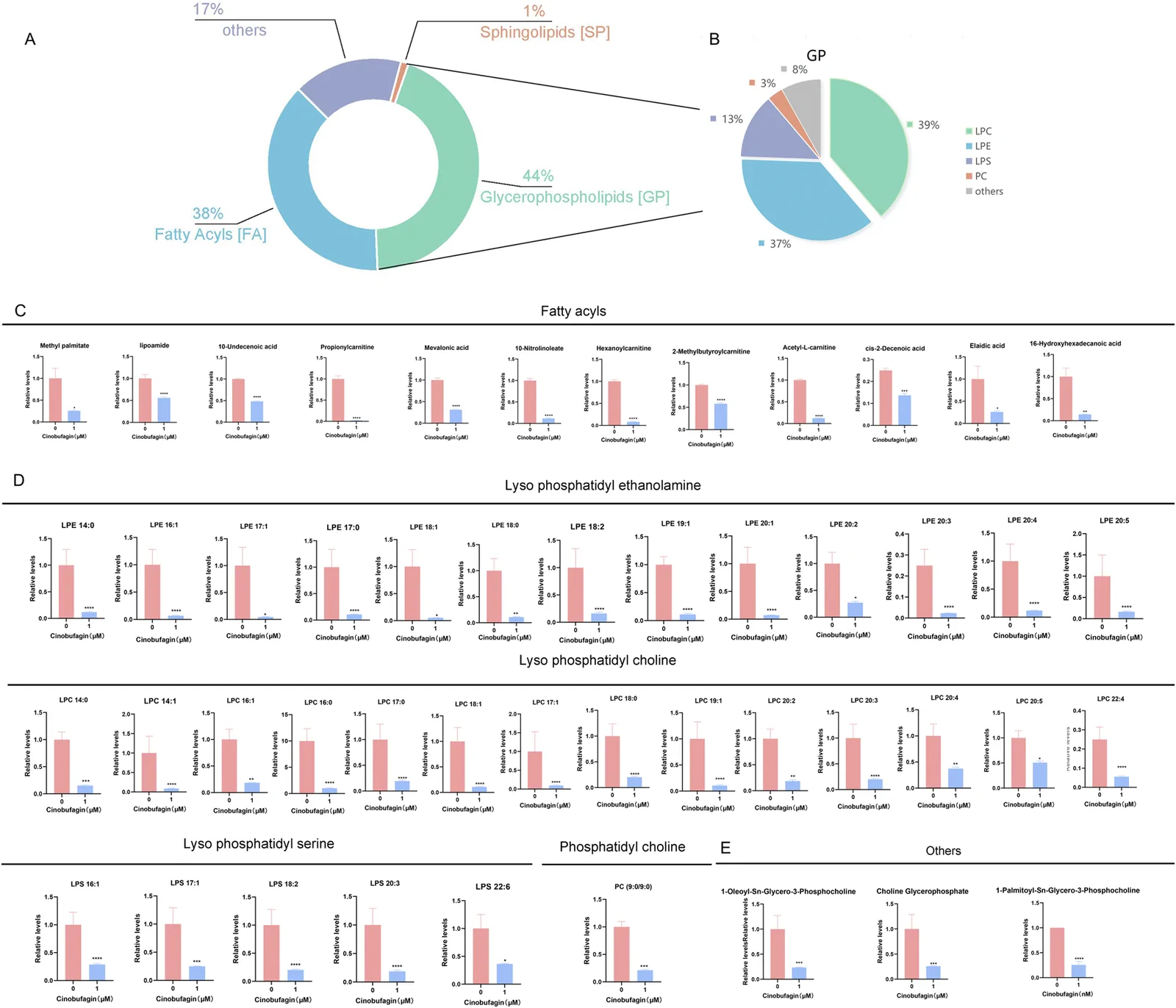
Cinobufagin suppresses the levels of fatty acyls and glycerophospholipids in HepG2 cells. **(A)** Proportion of lipid subclasses in cinobufagin-treated cells. **(B)** Proportion of each subclass of glycerophospholipids in cinobufagin-treated cells. **(C)** Levels of fatty acyls in HepG2 cells exposed to cinobufagin. **(D)** Levels of glycerophospholipids (LPE, LPC, LPS, and PC) in HepG2 cells exposed to cinobufagin. **(E)** Levels of other glycerophospholipids in HepG2 cells exposed to cinobufagin. ^*^ *p* < 0.05, ^**^ *p* < 0.01, ^***^ *p* < 0.001, ^****^ *p* < 0.0001.

### 3.7 Cinobufagin restrains the tumor growth in H22-hepatoma-bearing mice

In order to study the antitumor effects of cinobufagin *in vivo*, mouse H22 hepatoma cells were injected into the right posterior dorsal region of Kunming mice. As shown in Figure 9A, results showed that cinobufagin treatment could significantly reduce the tumorigenic ability of H22 cells in Kunming mice. No significant weight loss was observed in mice treated with cinobufagin, suggesting that the systemic toxicity of cinobufagin treatment is low (Figure 9B). In order to further study the potential toxicity of cinobufagin on normal tissues, the heart, liver, spleen, lung, and kidney of mice were collected and H&E staining was performed. As shown in Figure 9C, H&E staining showed no signs of toxicity to normal organ tissues in response to cinobufagin.

**FIGURE 9:**
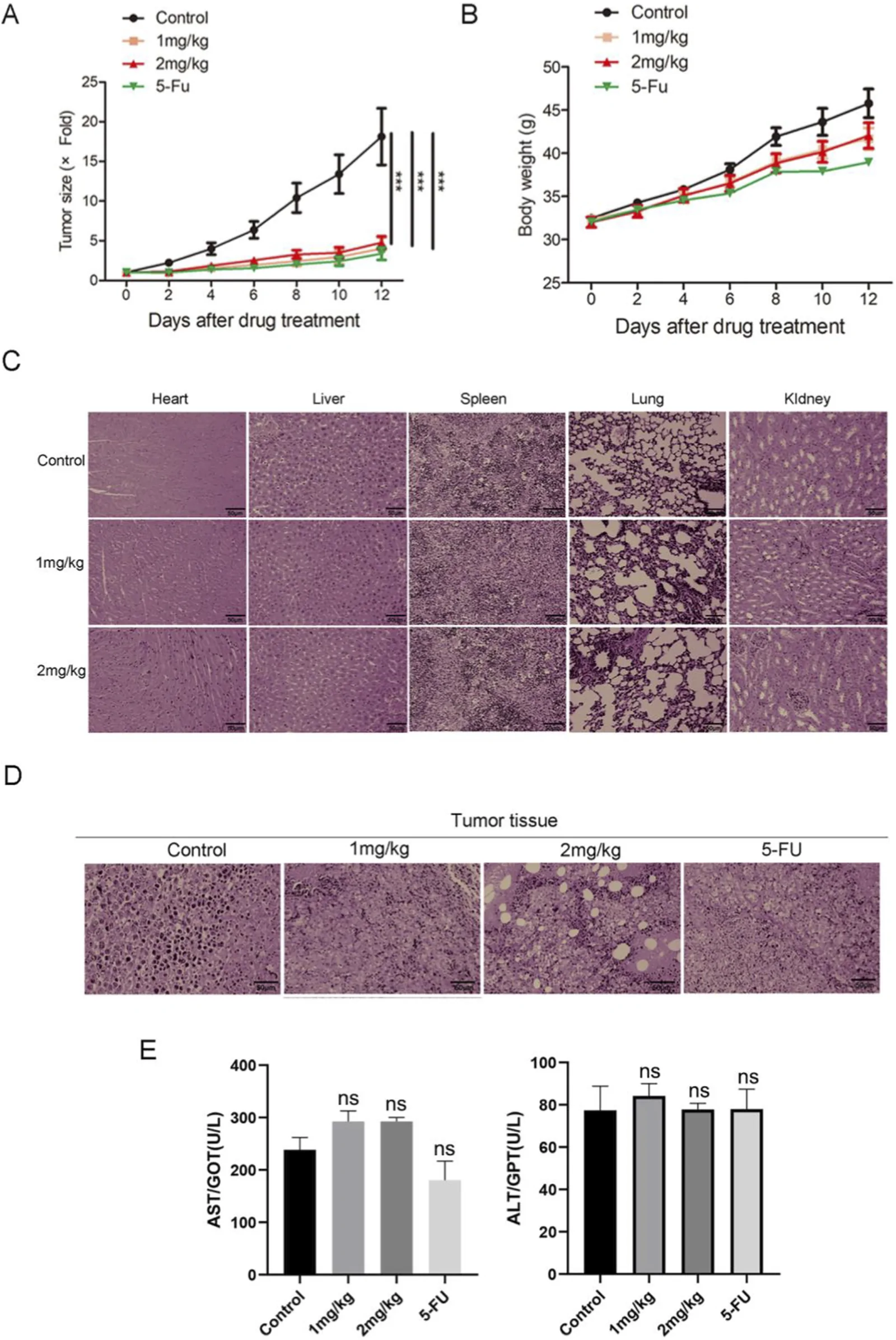
Cinobufagin suppresses the oncogenicity of mouse hepatoma H22 cells in Kunming mice. **(A)** Tumor growth fold curve. **(B)** Body weight of the mice during drug treatment, n = 10. **(C)** H&E staining of major organs from tumor-bearing mice in the control group or the drug-treatment group. Scale bar, 50 µm. **(D)** H&E staining of tumor tissues from tumor-bearing mice in the control group or the drug-treatment group. Scale bar, 50 µm. **(E)** Serum levels of ALT and AST in tumor-bearing mice in the control group or the drug-treatment group, n = 10. ^***^ *p* < 0.001.

Mouse tumor tissues were collected and stained with H&E. As shown in Figure 9D, tumor cells in the negative control group showed diffuse or clumpy growth. Tumor cells were closely arranged, cytoplasm was abundant, and nuclei were large and deeply stained. Tumor cells in the positive drug 5-FU group were sparsely arranged, had consolidated and ruptured nuclei, and exhibited more connective tissue proliferation. In the cinobufagin treatment group, tumor cells varied in size, with reduced cell density, sparse arrangement, nuclear contraction and rupture, and more connective tissue hyperplasia. In addition, with the increase of the dose of cinobufagin, the number of tumor cells showing necrosis increased, the nuclear coloring was not uniform, and the cancer cell tissues showed a clumpy shape and cancer nests. Blood samples were collected for serum biochemical analysis of alanine aminotransferase (ALT) and aspartate aminotransferase (AST). There was no significant difference in ALT and AST levels between the control group and cinobufagin treatment group, indicating that cinobufagin had no obvious adverse effects on liver function (Figure 9E).

The *in vivo* serum metabolomics data were also analyzed. First, a PLS-DA model containing control and cinobufagin-treated samples was established to observe the differences between these two groups. As shown in Figures 10A,B, PLS-DA analysis of metabolites measured by LC-MS (+) and LC-MS (−) showed significant differences in metabolic composition between the control group and the cinobufagin treatment group. In addition, the results of the arrangement test showed that when the red regression line of Q2 points below zero, the established PLS-DA model has high reliability and prediction ability, and there was no overfitting phenomenon (Figures 10C,D). Volcano maps generated by R language (Figures 10E,F) contain fold changes and *p*-values. Significantly downregulated metabolites are green, while upregulated metabolites are red. The classification of differential metabolites and the proportion of each part are shown in Figures 10G,H, in which GP and FA account for a large proportion. In addition, the matchstick map clearly exhibits the differential metabolites and the substances with large difference multiples (Figures 10I,J).

**FIGURE 10:**
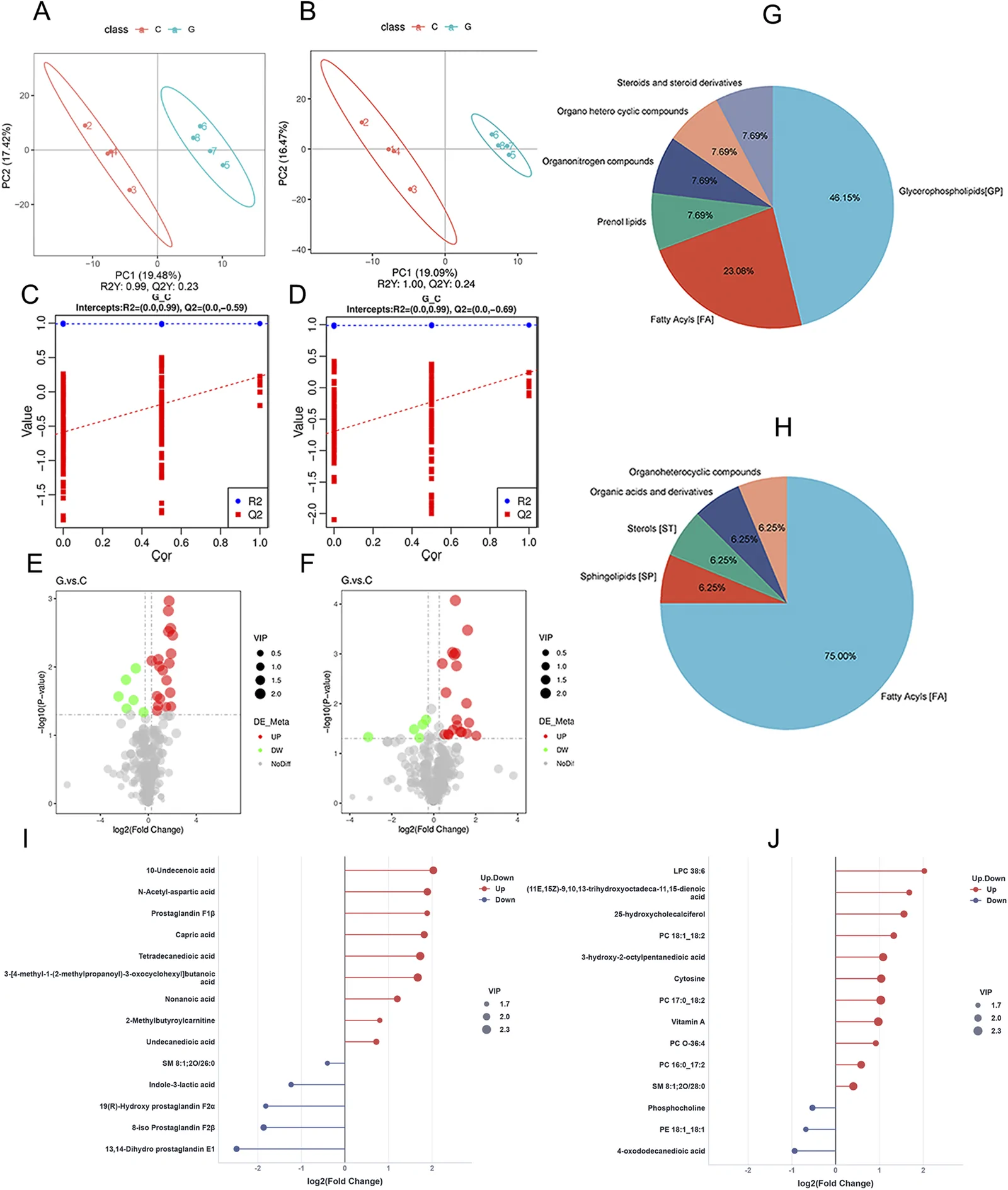
Metabolomics analysis of serum of hepatoma H22-bearing Kunming mice treated with or without cinobufagin. In **(A–F)**, C indicates the control group, and G indicates the high-dose cinobufagin treatment group. **(A,B)** Results of partial least squares discriminant (PLS-DA) analysis: **(A)** negative mode and **(B)** positive mode. **(C)** Negative-mode results of the permutation test. **(D)** Positive-mode results of the permutation test. **(E,F)** Volcano plots of the cinobufagin-treated group/control group: **(E)** negative mode and **(F)** positive mode. **(G,H)** Proportion of chemical classes among differential metabolites: **(G)** negative mode and **(H)** positive mode. **(I,J)** Matchstick plot of differential metabolites of the cinobufagin-treated group/control group: **(I)** negative mode and **(J)** positive mode. The length of the rod represents the magnitude of log_2_ (fold change). The color of the bar indicates whether the change represents up- or downregulation. Red represents upregulation, and blue represents downregulation. The size of the dots represents the magnitude of the variable importance in projection (VIP) values. The preliminary metabolic characteristics shown in the figure do not indicate consistent confidence for all metabolites.

A heat map can directly show the difference in the level of metabolites in each sample. The level of identified lipids and lipidoid differential metabolites was compared using heat maps (Figures 11A,B; Table 2). The data showed that 24 kinds of lipids and lipidoid molecules had significant changes in response to cinobufagin, including 14 fatty acids, six glycerophospholipids, one sphingolipid, two sterols, and one prenol lipid. Six lipids and lipidoid molecules were downregulated. In addition, as shown in Figure 11C and Table 3, two identified differential organic heterocyclic compounds, including 3-indole-lactic acid and cytosine, were downregulated after exposure to cinobufagin. The level of phosphocholine, a phospholipid headgroup component and glycerophospholipid metabolic intermediate, was significantly reduced after cinobufagin treatment (Figure 11D).

**FIGURE 11:**
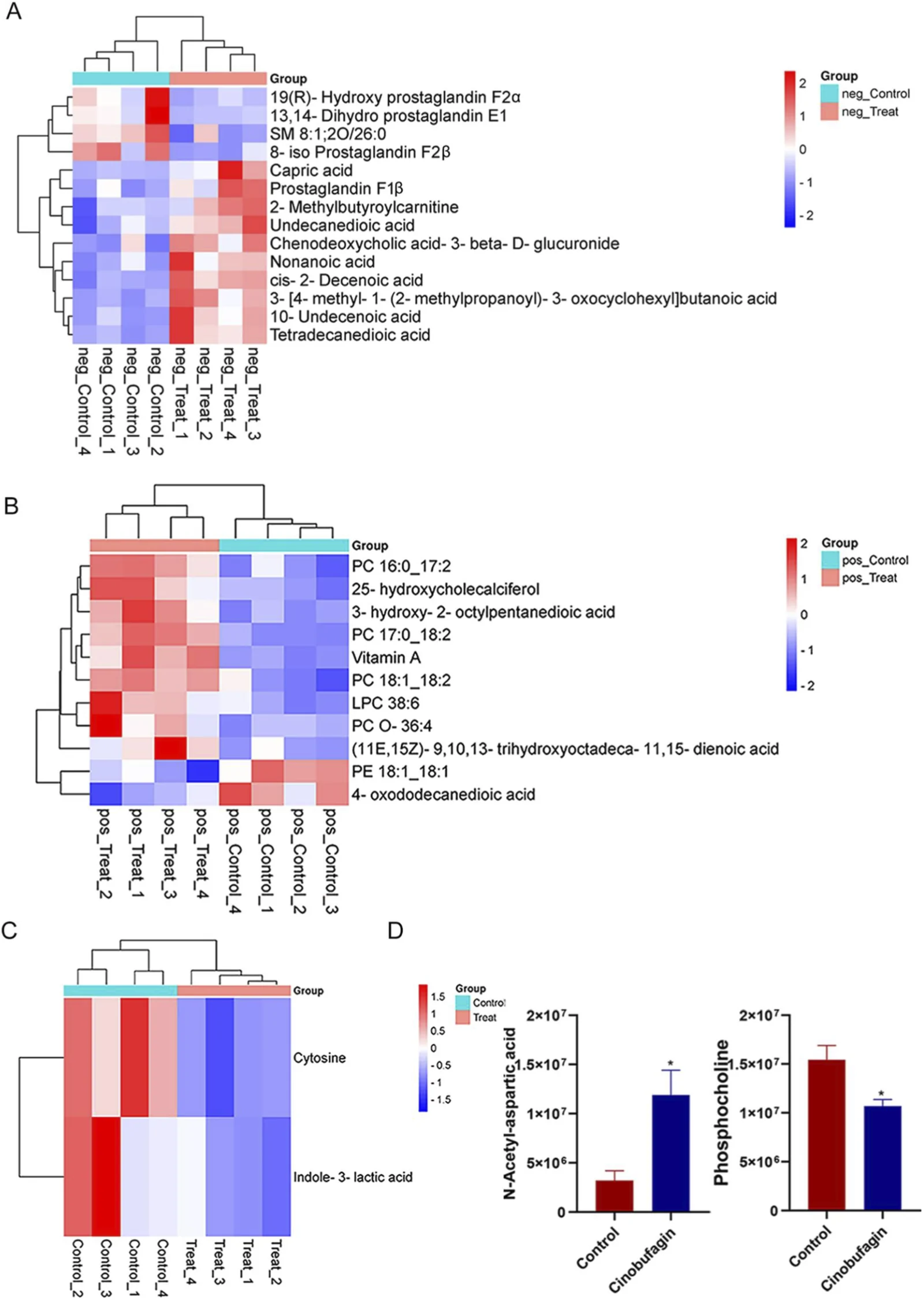
Heat map analysis of differential metabolites in the serum of hepatoma H22-bearing Kunming mice treated with cinobufagin. **(A,B)** Heat map analysis of lipid and lipid-like differential metabolites identified in serum of mice: **(A)** negative mode and **(B)** positive mode. Red represents high expression, and blue represents low expression. Each row represents a single metabolite. Each column represents one sample. **(C)** Heatmap analysis of differential organoheterocyclic metabolites identified in serum of mice. **(D)** Phosphocholine and N-acetyl-aspartic acid levels identified in serum of mice. ^*^ *P* < 0.05. The preliminary metabolic characteristics shown in the figure do not indicate consistent confidence for all metabolites.

**TABLE 2:** Details of identified 24 lipids and lipid-like differential metabolites in serum of mice treated with or without cinobufagin based on the Human Metabolome Database (HMDB) and the LIPID Maps database**.**

Category	Name (up)	Name (down)	Number
Fatty acyls [FA]	Prostaglandin F1	​	​
	Tetradecanedioic acid	8-Iso prostaglandin F2	Down (4), up (10)
	3-[4-Methyl-1-(2-methylpropanoyl)-3-oxocyclohexyl]butanoic acid	13,14-Dihydro prostaglandin E1	
	10-Undecenoic acid	​	
	Nonanoic acid	19(R)-Hydroxy prostaglandin F2	
	Capric acid	​	
	2-Methylbutyroylcarnitine	​	
	3-Hydroxy-2-octylpentanedioic acid	​	
	(11E,15Z)-9,10,13-Trihydroxyoctadeca-11,15-dienoic acid	​	
	Undecanedioic acid	4-Oxododecanedioic acid	
Glycerophospholipids [GP]	PC 17:0_18:2	PE 18:1_18:1	Down (1), up (5)
	PC 16:0_17:2	​	
	PC O-36:4	​	
	PC 18:1_18:2	​	
	LPC 38:6	​	
Sphingolipids [SP]	​	SM 8:1; 2O/26:0	Down (1)
Sterols [ST]	25-hydroxycholecalciferol	​	Up (2)
	Chenodeoxycholic acid-3-beta-D-glucuronide	​	
Prenol lipids	Vitamin A	​	Up (1)

**TABLE 3:** Details of differential organoheterocyclic metabolites in the serum of mice treated with or without cinobufagin based on the HMDB database**.**

Category	Name (Up)	Name (Down)	Number
Indoles and derivatives	​	Indole-3-lactic acid	Down (1)
Diazines	Cytosine	​	Up (1)

## 4 Discussion

Primary liver cancer is the sixth most common cancer worldwide (Grandhi et al., 2016). To date, there is still a lack of effective drugs to inhibit the progression of liver cancer, and there is an urgent need to develop new effective and low-toxicity anticancer drugs. At present, the search for new active substances from natural products is the focal point of anticancer drug research. The main bioactive chemical components of Chansu have been identified as bufadienolides, indole alkaloids, and polypeptides. Bufadienolides are the major active ingredients, including cinobufagin, bufalin, and others. Bufadienolides have been proven to exhibit excellent antitumor activity. Studies have reported that cardiac glycoside natural products (including certain bufadienolides) can enhance tumor cell sensitivity to radiotherapy and chemotherapy by inhibiting DNA double-strand break repair (Surovtseva et al., 2016). Yuan et al. (2016) reported that active bufadienolide compounds can affect the proportion of CD4^+^CD25^+^Foxp3^+^regulatory T cells in mitogen-activated human peripheral blood mononuclear cells, suggesting their potential involvement in tumor immune regulation. Nogawa et al. (2001) isolated and identified five new bufadienolides from Chansu and confirmed their growth-inhibitory activity. Moreno et al. (2013), through structure-activity relationship analysis, systematically investigated the *in vitro* growth-inhibitory effects of 27 bufadienolides on mouse and human cancer cells, revealing the impact of structural modifications on activity. Meng et al. (2024) isolated multiple structurally diverse bufadienolides from the skin of *Bufo bufo* gargarizans and evaluated their cytotoxicity. Somani et al. (2025) explored the anticancer properties of synthetic glycan analogs of proscillaridin A. Xiaolu Wei et al. (2017) confirmed bufadienolides as the main antitumor components in cinobufacin injection for the treatment of liver and gastric cancer. Other studies have revealed that this class of compounds can exert antitumor effects by inducing cell cycle arrest, apoptosis, and DNA damage, inhibiting Na^+^/K^+_^ ATPase, as well as regulating signaling pathways such as PI3K/AKT/ERK/Snail and p53 (Lan et al., 2018; Lin et al., 2018; Gao et al., 2018).

Cinobufagin, as a bufadienolide, is a key antitumor active component of Chansu (Yang et al., 2021). Published studies have shown that cinobufagin restrains the growth of human liver cancer cells and triggers DNA damage through proteasome-dependent degradation of thymidylate synthase (Yang et al., 2022). It has been shown that bufalin and cinobufagin induce apoptosis in human hepatoma cells through Fas and mitochondrial pathways (Qi et al., 2011).

The effects of cinobufagin on peroxisomes have not been reported. Compared with the previously reported mechanisms, the present study reveals for the first time that cinobufagin induces peroxisomal dysfunction and interferes with lipid metabolism by suppressing proteins encoded by PEX genes, thereby exerting antitumor effects. 

Peroxisomes are multifunctional organelles present in almost all eukaryotic cells. They were first discovered in 1954 and formalized and named as an organelle in 1966 (De Duve and Baudhuin, 1966). Peroxisomes are often thought of as “cellular hoi polloi” that are used to scavenge reactive oxygen chemical debris discarded by other organelles. However, their function goes far beyond the metabolism of hydrogen peroxide. The occurrence of peroxisomes depends mainly on a variety of PEX proteins encoded by different PEX genes. Mutations in the PEX gene and abnormal function of the PEX protein will affect the division and proliferation of peroxisomes (Braverman et al., 2013). Many recent studies have shown that peroxisomes play a crucial role in cancers characterized by metabolic abnormalities, such as liver cancer and colorectal cancer (Kim, 2020; Cai et al., 2018). Enzymes involved in the processing of peroxisome lipids are elevated in many types of tumors (Benjamin et al., 2013). Moreover, different types of tumors exhibit a reduction in growth when the expression of genes associated with peroxisomal degradation is regulated, or peroxisomal lipid processing is inhibited in mice (Dahabieh et al., 2022; Lee et al., 2025; Mah et al. 2024). This suggests a potential role for targeting peroxisomal pathways as a potential treatment option for various types of cancer.

Human peroxisomes contain proteins including the peroxins (PEX), which are involved in peroxisome biogenesis; ABCD transporters (ABCD1, ABCD2, and ABCD3), which import fatty acids into the peroxisome that then undergo a process of synthesis or degradation through β-oxidation; and several matrix enzymes which catalyze these processes. The PEX5 gene, located on chromosome 12p13.31, encodes a Type 1 peroxisome targeting signal receptor (PTS1R) that is required for peroxisome biogenesis. Most enzymes in the peroxisome are transported from the cytosol with the aid of the PEX5 receptor, which binds to an anchoring complex on the peroxisome membrane. Upon completion of transport, the ubiquitin ligase (PEX2, PEX10, and PEX12) on the PEX5 coating adds a single ubiquitin molecule, followed by exportomer-mediated (PEX1, PEX6, and PEX26) removal of PEX5 from the membrane, and recycling of PEX5 back to the cytosol (Dahabieh et al., 2018).

Peroxisome biogenesis is a complex multi-step process, in which PEX5, PEX10, and PEX6 play consecutive and mutually non-replaceable roles. PEX5 acts as a cytoplasmic receptor, responsible for recognizing matrix proteins in the cytoplasm that carry peroxisome targeting signal 1 (PTS1) and transporting them to the peroxisome membrane. PEX10 serves as an essential component of the membrane-bound RING-type ubiquitin ligase complex and mediates the ubiquitination of PEX5, a critical step in the recycling process of the PEX5 receptor. PEX6, a cytosolic AAA + ATPase, acts downstream in this pathway. It hydrolyzes ATP to release energy, thereby facilitating the dissociation of ubiquitinated PEX5 from the peroxisomal membrane and its subsequent recycling to the cytoplasm (Farré et al., 2019). It can be seen that the functional differences explain why disrupting different nodes in the pathway can lead to different reactions.

PEX5 plays an important role in peroxisome function, encoding proteins that regulate REDOX homeostasis during intercellular stress (Wang and Subramani, 2017). Studies have shown that cancer cell proliferation is inhibited after PEX5 or PEX10 deletion (Cai et al., 2018). It has been reported that high expression of PEX5 suggests an association with poor prognosis in HCC patients, and ectopic expression of PEX5 increases proliferation, migration, and invasive ability of cancer cells (Wen et al., 2020). Targeting PEX5 or other PEX genes may be an effective strategy for liver cancer therapy.

In our study, we show that cinobufagin causes peroxisome dysfunction and reduces the number of peroxisomes in HepG2 and SK-HEP-1 tumor cells. Moreover, based on the hints of transcriptomic results, we identify that cinobufagin inhibits the expression of PEX5, PEX6, and PEX10 in HepG2 and SK-HEP-1 tumor cells. Knockdown of PEX5 or PEX10 inhibits the colony-forming ability and proliferation of HepG2 and SK-HEP-1 tumor cells. Suppression of PEX5 or PEX10 could enhance the inhibitory effects of cinobufagin on the proliferative potential of cancer cells. It is noteworthy that although cinobufagin downregulated PEX6 expression, knocking down PEX6 did not significantly affect the proliferation of HepG2 and SK-HEP-1 cells. This discrepancy may be related to functional specificity among members of the PEX protein family or cell type specificity. The biological significance of cinobufagin’s inhibition of PEX6 in this study may not lie in suppressing tumor cell proliferation.

The peroxisome plays an important role in many lipid-related processes. Defects in genes coding for peroxisome proteins have been linked to peroxisome diseases, many of which exhibit inherent metabolic errors. Abnormal lipid metabolism has also become the hallmark of malignant tumors (Bian et al., 2021). Published reports have demonstrated that abnormal lipid metabolism occurs in a variety of tumors, including HCC, and is characterized by accelerated lipid uptake and biosynthesis (Fhaner et al., 2012; Wong et al., 2025). Reprogramming of lipid metabolism also plays an important role in the growth and metastasis of liver cancer cells (Wong et al., 2025). Modulating lipid metabolic processes can be a potential candidate strategy for clinical cancer therapy.

Liver cancer is typically characterized by upregulation of genes encoding key enzymes involved in fatty acid synthesis, such as ACLY and FASN (Cai et al., 2018). The results of our study reveal that cinobufagin inhibits the expression of ACLY and FASN proteins, which may induce inhibition of fatty acid synthesis. Moreover, cinobufagin treatment also inhibits the expression of key fatty acid oxidation (FAO)-related enzyme ECH1. Further mechanistic investigation demonstrates that knockdown of PEX5 inhibits the expression of FASN and ACLY in HepG2 and SK-HEP-1 tumor cells, and depletion of PEX10 restrains FASN levels in cancer cells. These results imply that cinobufagin restrains the expression of lipid metabolism-associated proteins partially through suppression of PEX5 and PEX10, moreover, cinobufagin may interfere with lipid metabolic reprogramming in HepG2 and SK-HEP-1 tumor cells.

Using metabolomics technology, 47 lipid-related metabolites were discovered to be downregulated in HepG2 cells in response to cinobufagin: 12 metabolites of fatty acyl, glycerophospholipids including 13 metabolites of LPE, 14 metabolites of LPC, and 4 metabolites of LPS, 1 metabolite of PC, and 3 other glycerophospholipids. Research reports have demonstrated that both LPC and LPE are elevated in HCC (Wang et al., 2012). LPC has been reported to stimulate migration of tumor cells through their respective G-protein coupled receptors (Gaetano et al., 2009). LPE has been identified to promote the migration and proliferation of tumor cells *via* activation of MAPK signaling. LPS stimulates chemotactic migration of tumor cells through GPR34 and the PI3K/AKT pathway (Park et al., 2014; Iida et al., 2014). The suppression of key enzymes related to lipid metabolism may be partially responsible for the inhibition of fatty acyl and glycerophospholipids in response to cinobufagin.

*In vivo* experiments show that cinobufagin could attenuate the tumorigenic ability of H22 cells in Kunming mice without obvious side effects. *In vivo* serum metabolomics data show that cinobufagin mainly causes significant changes in the level of lipids and lipid-like metabolites. Under cinobufagin treatment, 24 lipids and lipidoid molecules are significantly changed, including 14 fatty acyls, six glycerophospholipids, one sphingolipid, two sterols, and one prenol lipid. The downregulated differential metabolites of lipids and lipidoid molecules include iso 8-prostaglandin F2β, 19(R)-hydroxy prostaglandin F2α, SM 8:1; 2O/26:0, 4-oxododecanedioic acid, 13,14-dihydro prostaglandin E1, and phosphatidyl ethanolamine (PE) 18:1_18:1. In addition, 17 differential metabolites of lipids and lipidoid molecules are upregulated after cinobufagin treatment.

Studies in recent years have found that abnormalities in lipid metabolism are closely related to the development of cancer. Some studies have shown that in cancer cells, fatty acids enter the peroxisome more often (Gao et al., 2020). Sphingolipids are a group of amphipathic lipids containing a sphingomyelin backbone with a long chain of fatty acids attached to one end and a polar alcohol at the other. Sphingolipids include sphingomyelins, gangliosides, and others. Disorders of sphingolipid metabolism can induce a variety of diseases, including HCC (Canals et al., 2011; Li et al., 2018). Studies have shown that sphingolipids can be used as a new marker for the diagnosis of HCC, and the regulation of sphingolipid metabolism may become a potential strategy for the treatment of HCC (Jiang and Zheng, 2018). Alexia et al. found that SM 18:2/24:1 is significantly increased in patients with HCC (Cotte et al., 2019). In the present study, cinobufagin reduces the level of SM 8:1; 2O/26:0 in the serum of hepatoma H22-bearing Kunming mice.

Glycerophospholipids (GP) are the most abundant phospholipids in the body. In addition to forming biological membranes, GP is also one of the components of bile and membrane surfactants and participates in the recognition and signal transduction of proteins by cell membranes. Phosphatidyl ethanolamine (PE), a type of glycerophospholipid, is also known as ethanolamine phosphoglycerate, which is a phospholipid compound consisting of glycerol, fatty acids, phosphoric acid, and ethanolamine. Dysregulation of PE levels is frequently observed in cancer. Studies have shown that serum levels of phosphatidylethanolamine (PE) are significantly elevated in patients with hepatocellular carcinoma compared to those with liver cirrhosis (Rashid et al., 2023). In the present study, we find that cinobufagin can reduce PE 18:1_18:1 levels in hepatoma H22-bearing Kunming mice.

Sterols (ST) include cholesterol and its derivatives, which are important components of biological membranes. The secosteroid, which includes many different forms of vitamin D, is a mammalian derivative of oxidized cholesterol and is produced in the liver. Our study reveals that cinobufagin can upregulate the levels of sterol metabolites chenodeoxycholic acid-3-beta-D-glucuronide and 25-hydroxycholecalciferol in hepatoma H22-bearing Kunming mice. Chenodeoxycholic acid has been reported as a serum metabolite biomarker for HCC diagnosis in previous studies (Liu et al., 2013). Studies have shown low levels of the bile-acid-metabolism-related metabolite (chenodeoxycholic acid) in HCC tissues compared to non-cancerous tissues (Han et al., 2019). 25-Hydroxycholecalciferol (25-hydroxyvitamin D3) is a prohormone derived from the hydroxylation of vitamin D3 in the liver. Vitamin D has been associated with a reduced risk of several cancers. Vitamin D has been shown to inhibit cellular proliferation and induce differentiation and apoptosis in malignant breast cells (Crew et al., 2009). A previous study revealed that 25-hydroxycholecalciferol deficiency is associated with an advanced stage of HCC and is a poor prognostic indicator (Finkelmeier et al., 2014). Prenol lipids and their phosphorylated derivatives have an important role in the transport of oligosaccharides between cell membranes. Studies have suggested that low vitamin A levels may be associated with the risk of hepatitis C, cirrhosis, and HCC (Peres et al., 2011). Some studies have reported that vitamin A inhibits the metabolic activity of hepatic microsomal enzymes on benzopyrene and prevents the transformation of carcinogens (Zhang et al., 2018). In our study, cinobufagin is found to increase the levels of the prenol lipid-like substance vitamin A in hepatoma H22-bearing Kunming mice.

In conclusion, cinobufagin induces differential changes in lipids and lipid-like substances in the serum of hepatoma H22-bearing Kunming mice, suggesting that cinobufagin may exert anticancer effects by regulating lipid metabolism. In addition, we also identify that cinobufagin can affect the levels of organoheterocyclic compounds such as indole-3-lactic acid and cytosine in the serum of hepatoma H22-bearing Kunming mice. In detail, the level of indole-3-lactic acid is downregulated. Serum levels of indole-3-lactic acid were found to be significantly higher in patients with lung cancer compared to healthy controls (Sun et al., 2019). Cinobufagin significantly reduced the serum level of phosphocholine in tumor-bearing mice. Choline metabolites in tumors are characterized by elevated phosphocholine and total choline compounds (Glunde and Serkova, 2006).

## 5 Conclusion

In summary, our results indicate that cinobufagin leads to peroxisome dysfunction in liver cancer cells. Through further mechanism studies, results show that cinobufagin suppresses the peroxisome proteins PEX5, PEX6, and PEX10. We demonstrate that cinobufagin restrains lipid metabolism-related key enzymes FASN, ACLY, and ECH1. More importantly, we also identify that cinobufagin restrains the expression of lipid metabolism-associated proteins partially through suppression of PEX5 and PEX10. In addition, depletion of PEX5 or PEX10 could restrain the proliferation and colony formation of liver cancer cells and enhance the inhibitory effect of cinobufagin on the proliferative potential of tumor cells, suggesting that cinobufagin exhibits anti-hepatoma activity *in vitro*, at least in part, by down-regulating PEX5 or PEX10. Based on the results of peroxisome dysfunction and the intervention of protein expression related to lipid metabolism exposed to cinobufagin, metabolomics analysis results further identify that cinobufagin suppresses multiple kinds of fatty acyls and glycerophospholipids. *In vivo*, we demonstrate that cinobufagin restrains tumor growth in H22 tumor-bearing mice without obvious side effects. *In vivo* metabolomics analysis shows that cinobufagin could exert antitumor effects by affecting lipid metabolism.

Our study suggests that peroxisome proteins may be potential molecular targets for liver cancer treatment. This work lays a theoretical foundation for the clinical application of cinobufagin and Chansu and promotes the development of new drugs from TCM.

## Data Availability

The original contributions presented in the study are included in the article/supplementary material, further inquiries can be directed to the corresponding author.

## References

[B1] Abdel-Magid A. F. (2015). Fatty acid synthase (FASN) inhibitors as potential treatment for cancer, obesity, and liver related disorders. ACS Med. Chem. Lett. 6 (8), 838–839. 10.1021/acsmedchemlett.5b00275 26288680 PMC4538450

[B3] Benjamin D. I. Cozzo A. Ji X. Roberts L. S. Louie S. M. Mulvihill M. M. (2013). Ether lipid generating enzyme AGPS alters the balance of structural and signaling lipids to fuel cancer pathogenicity. Proc. Natl. Acad. Sci. U. S. A. 110 (37), 14912–14917. 10.1073/pnas.1310894110 23980144 PMC3773741

[B58] Bian X. Liu R. Meng Y. Xing D. Xu D. Lu Z. (2021). Lipid metabolism and cancer. J. Exp. Med. 218 (1), e20201606. 10.1084/jem.20201606 33601415 PMC7754673

[B5] Braverman N. E. D'Agostino M. D. Maclean G. E. (2013). Peroxisome biogenesis disorders: biological, clinical and pathophysiological perspectives. Dev. Disabil. Res. Rev. 17 (3), 187–196. 10.1002/ddrr.1113 23798008

[B7] Brown A. I. Kim P. K. Rutenberg A. D. (2014). PEX5 and ubiquitin dynamics on Mammalian peroxisome membranes. PLoS Comput. Biol. 10 (1), e1003426. 10.1371/journal.pcbi.1003426 24453954 PMC3894153

[B8] Bruix J. Llovet J. M. (2002). Prognostic prediction and treatment strategy in hepatocellular carcinoma. Hepatology 35 (3), 519–524. 10.1053/jhep.2002.32089 11870363

[B9] Cai M. Sun X. Wang W. Lian Z. Wu P. Han S. (2018). Disruption of peroxisome function leads to metabolic stress, mTOR inhibition, and lethality in liver cancer cells. Cancer Lett. 421, 82–93. 10.1016/j.canlet.2018.02.021 29458144

[B10] Canals D. Perry D. M. Jenkins R. W. Hannun Y. A. (2011). Drug targeting of sphingolipid metabolism: sphingomyelinases and ceramidases. Br. J. Pharmacol. 163 (4), 694–712. 10.1111/j.1476-5381.2011.01279.x 21615386 PMC3111673

[B11] Chen X. Cheung S. T. So S. Fan S. T. Barry C. Higgins J. (2002). Gene expression patterns in human liver cancers. Mol. Biol. Cell. 13 (6), 1929–1939. 10.1091/mbc.02-02-0023 12058060 PMC117615

[B72] Corbett V. Li D. Chauhan A. (2023). Review of practice informing data and current state of NCCN consensus guidelines in hepatobiliary cancers. Hepatobil. Surg. Nutr. 12 (5), 798–803. 10.21037/hbsn-23-372 37886197 PMC10598301

[B73] Cotte A. K. Cottet V. Aires V. Mouillot T. Rizk M. Vinault S. (2019). Phospholipid profiles and hepatocellular carcinoma risk and prognosis in cirrhotic patients. Oncotarget 10 (22), 2161–2172. 10.18632/oncotarget.26738 31040908 PMC6481329

[B13] Crew K. D. Gammon M. D. Steck S. E. Hershman D. L. Cremers S. Dworakowski E. (2009). Association between plasma 25-hydroxyvitamin D and breast cancer risk. Cancer Prev. Res. 2 (6), 598–604. 10.1158/1940-6207.CAPR-08-0138 19470790 PMC3077714

[B14] Dahabieh M. S. Di Pietro E. Jangal M. Goncalves C. Witcher M. Braverman N. E. (2018). Peroxisomes and cancer: the role of a metabolic specialist in a disease of aberrant metabolism. Biochim. Biophys. Acta-Rev. Cancer 1870 (1), 103–121. 10.1016/j.bbcan.2018.07.004 30012421

[B90] Dahabieh M. S. Huang F. Goncalves C. Flores González R. E. Prabhu S. Bolt A. (2022). Silencing PEX26 as an unconventional mode to kill drug-resistant cancer cells and forestall drug resistance. Autophagy 18, 540–558. 10.1080/15548627.2021.1936932 PMC9037551 34074205

[B15] De Duve C. Baudhuin P. (1966). Peroxisomes (microbodies and related particles). Physiol. Rev. 46 (2), 323–357. 10.1152/physrev.1966.46.2.323 5325972

[B61] Fan S. Guo J. Nie H. Xiong H. Xia Y. (2024). Aberrant energy metabolism in tumors and potential therapeutic targets. Genes Chromosomes Cancer 63 (11), e70008. 10.1002/gcc.70008 PMC11587691 39584783

[B93] Farré J.-C. Mahalingam S. S. Proietto M. Subramani S. (2019). Peroxisome biogenesis, membrane contact sites, and quality control. EMBO Rep. 20, e46864. 10.15252/embr.201846864 30530632 PMC6322382

[B83] Farré J.-C. Mahalingam S. S. Proietto M. Subramani S. (2019). Peroxisome biogenesis, membrane contact sites, and quality control. EMBO Rep. 20, e46864. 10.15252/embr.201846864 30530632 PMC6322382

[B16] Fhaner C. J. Liu S. Ji H. Simpson R. J. Reid G. E. (2012). Comprehensive lipidome profiling of isogenic primary and metastatic Colon adenocarcinoma cell lines. Anal. Chem. 84 (21), 8917–8926. 10.1021/ac302154g 23039336 PMC3491142

[B17] Finkelmeier F. Kronenberger B. Koberle V. Bojunga J. Zeuzem S. Trojan J. (2014). Severe 25-hydroxyvitamin D deficiency identifies a poor prognosis in patients with hepatocellular carcinoma - a prospective cohort study. Aliment. Pharmacol. Ther. 39 (10), 1204–1212. 10.1111/apt.12731 24684435

[B18] Gaetano C. G. Samadi N. Tomsig J. L. Macdonald T. L. Lynch K. R. Brindley D. N. (2009). Inhibition of autotaxin production or activity blocks lysophosphatidylcholine-induced migration of human breast cancer and melanoma cells. Mol. Carcinog. 48 (9), 801–809. 10.1002/mc.20524 19204929 PMC2736327

[B19] Gao Y. Shi L. Cao Z. Zhu X. Li F. Wang R. (2018). Telocinobufagin inhibits the epithelial-mesenchymal transition of breast cancer cells through the phosphoinositide 3-kinase/protein kinase B/extracellular signal-regulated Kinase/snail signaling pathway. Oncol. Lett. 15 (5), 7837–7845. 10.3892/ol.2018.8349 29725474 PMC5920466

[B20] Gao G. Sheng Y. Zhang J. Liu J. Xu L. (2020). Bodipy-C12 was enriched in peroxisomes of cancer cells. Mod. Biomedical Advances 20 (01), 1–7.

[B21] Glunde K. Serkova N. J. (2006). Therapeutic targets and biomarkers identified in cancer choline phospholipid metabolism. Pharmacogenomics 7 (7), 1109–1123. 10.2217/14622416.7.7.1109 17054420

[B22] Grandhi M. S. Kim A. K. Ronnekleiv-Kelly S. M. Kamel I. R. Ghasebeh M. A. Pawlik T. M. (2016). Hepatocellular carcinoma: from diagnosis to treatment. Surg. Oncol. 25 (2), 74–85. 10.1016/j.suronc.2016.03.002 27312032

[B23] Han J. Qin W. X. Li Z. L. Xu A. J. Xing H. Wu H. (2019). Tissue and serum metabolite profiling reveals potential biomarkers of human hepatocellular carcinoma. Clin. Chim. Acta. 488, 68–75. 10.1016/j.cca.2018.10.039 30389456

[B74] Hu B. Lin J. Z. Yang X. B. Sang X. T. (2020). Aberrant lipid metabolism in hepatocellular carcinoma cells as well as immune microenvironment: a review. Cell Prolif. 53 (3), e12772. 10.1111/cpr.12772 32003505 PMC7106960

[B24] Iida Y. H T. N. Kishikawa J. Kaneko K. Murono K. Kawai K. (2014). Lysophosphatidylserine stimulates chemotactic migration of colorectal cancer cells through GPR34 and PI3K/Akt pathway. Anticancer Res. 34 (10), 5465–5472. 25275042

[B25] Ikeda M. Morizane C. Ueno M. Okusaka T. Ishii H. Furuse J. (2018). Chemotherapy for hepatocellular carcinoma: current status and future perspectives. Jpn. J. Clin. Oncol. 48 (2), 103–114. 10.1093/jjco/hyx180 29253194

[B26] Jain I. H. Calvo S. E. Markhard A. L. Skinner O. S. To T. L. Ast T. (2020). Genetic screen for cell fitness in high or low oxygen highlights mitochondrial and lipid metabolism. Cell. 181 (3), 716–727.e11. 10.1016/j.cell.2020.03.029 PMC7293541 32259488

[B27] Jiang Y. Zheng S. (2018). Progress in research of sphingolipids in hepatocellular carcinoma. World Chin. J. Dig. 26 (36), 2109–2114. 10.11569/wcjd.v26.i36.2109

[B28] Kim J. A. (2020). Peroxisome metabolism in cancer. Cells 9 (7), 1692. 10.3390/cells9071692 32674458 PMC7408135

[B29] Lally J. Ghoshal S. Deperalta D. K. Moaven O. Wei L. Masia R. (2019). Inhibition of Acetyl-CoA carboxylase by phosphorylation or the inhibitor ND-654 suppresses lipogenesis and hepatocellular carcinoma. Cell. Metab. 29 (1), 174–182.e5. 10.1016/j.cmet.2018.08.020 30244972 PMC6643297

[B30] Lan Y. Wang X. Lou J. Xing J. Yu Z. Wang H. (2018). Bufalin inhibits glioblastoma growth by promoting proteasomal degradation of the Na(+)/K(+)-ATPase α1 subunit. Biomed. and Pharmacotherapy = Biomedecine and Pharmacotherapie 103, 204–215. 10.1016/j.biopha.2018.04.030 29653366

[B64] Lee H. Kang M. Sim S. H. Kang J. H. Choi W. Chun J. W. (2025). ACAA1 knockout increases the survival rate of KPC mice by activating autophagy. Mol. Metab. 100, 102237. 10.1016/j.molmet.2025.102237 PMC12419109 40848971

[B32] Li G. Liu D. Kimchi E. T. Kaifi J. T. Qi X. Manjunath Y. (2018). Nanoliposome C6-Ceramide increases the anti-tumor immune response and slows growth of liver tumors in mice. Gastroenterology 154 (4), 1024–1036.e9. 10.1053/j.gastro.2017.10.050 29408569 PMC5908238

[B65] Liang J.-J. Zhou X.-F. Long H. Li C.-Y. Wei J. Yu X.-Q. (2024). Recent advance of ATP citrate lyase inhibitors for the treatment of cancer and related diseases. Bioorg. Chem. 142, 106933. 10.1016/j.bioorg.2023.106933 37890210

[B33] Lin S. Lv J. Peng P. Cai C. Deng J. Deng H. (2018). Bufadienolides induce p53-mediated apoptosis in esophageal squamous cell carcinoma cells *in vitro* and *in vivo* . Oncol. Lett. 15 (2), 1566–1572. 10.3892/ol.2017.7457 29434851 PMC5774392

[B34] Liu S. Y. Zhang R. L. Kang H. Fan Z. J. Du Z. (2013). Human liver tissue metabolic profiling research on hepatitis B virus-related hepatocellular carcinoma. World J. Gastroenterol. 19 (22), 3423–3432. 10.3748/wjg.v19.i22.3423 23801834 PMC3683680

[B35] Llovet J. M. Kelley R. K. Villanueva A. Singal A. G. Pikarsky E. Roayaie S. (2021). Hepatocellular carcinoma. Nat. Rev. Dis. Prim. 7 (1), 6. 10.1038/s41572-020-00240-3 PMC13537433 33479224

[B36] Lodhi I. J. Semenkovich C. F. (2014). Peroxisomes: a nexus for lipid metabolism and cellular signaling. Cell. Metab. 19 (3), 380–392. 10.1016/j.cmet.2014.01.002 24508507 PMC3951609

[B37] Ma K. Zhang C. Huang M. Y. Li W. Y. Hu G. Q. (2016). Cinobufagin induces autophagy-mediated cell death in human osteosarcoma U2OS cells through the ROS/JNK/p38 signaling pathway. Oncol. Rep. 36 (1), 90–98. 10.3892/or.2016.4782 27176794 PMC4899018

[B66] Mah C. Y. Nguyen A. D. T. Niijima T. Helm M. Dehairs J. Ryan F. J. (2024). Peroxisomal β-oxidation enzyme, DECR2, regulates lipid metabolism and promotes treatment resistance in advanced prostate cancer. Br. J. Cancer 130, 741–754. 10.1038/s41416-023-02557-8 38216720 PMC10912652

[B39] Meng L. Cao Q. Du X. Lv H. Li C. Zhang X. (2024). Structurally diverse bufadienolides from the skins of bufo Bufo gargarizans and their cytotoxicity. Sci. Rep. 14 (1), 27344. 10.1038/s41598-024-79194-5 39521925 PMC11550443

[B40] Moreno L. Urban E. Gelbcke M. Dufrasne F. O. Kopp B. Kiss R. (2013). Structure-activity relationship analysis of bufadienolide-induced *in vitro* growth inhibitory effects on mouse and human cancer cells. J. Nat. Prod. 76 (6), 1078–1084. 10.1021/np400034d 23706005

[B41] Nagotu S. Kalel V. C. Erdmann R. Platta H. W. (2012). Molecular basis of peroxisomal biogenesis disorders caused by defects in peroxisomal matrix protein import. Biochim. Biophys. Acta 1822 (9), 1326–1336. 10.1016/j.bbadis.2012.05.010 22617146

[B43] Nogawa T. Kamano Y. Yamashita A. Pettit G. R. (2001). Isolation and structure of five new cancer cell growth inhibitory bufadienolides from the Chinese traditional drug Ch'an Su. J. Nat. Prod. 64 (9), 1148–1152. 10.1021/np0101088 11575946

[B67] Nuzzo P. V. Rodrigues S. Ribeiro C. F. Teixeira I. F. Fanelli G. N. Bleve S. (2025). Targeting cancer metabolism: therapeutic potential of the fatty acid synthase (FASN) inhibitors. Crit. Rev. Oncol. Hematol. 214, 104910. 10.1016/j.critrevonc.2025.104910 40846043 PMC13688562

[B44] Omata M. Cheng A. L. Kokudo N. Kudo M. Lee J. M. Jia J. (2017). Asia-pacific clinical practice guidelines on the management of hepatocellular carcinoma: a 2017 update. Hepatol. Int. 11 (4), 317–370. 10.1007/s12072-017-9799-9 28620797 PMC5491694

[B45] Park S. J. Lee K. P. Im D. S. (2014). Action and signaling of lysophosphatidylethanolamine in MDA-MB-231 breast cancer cells. Biomol. Ther. 22 (2), 129–135. 10.4062/biomolther.2013.110 24753818 PMC3975480

[B46] Peres W. A. Chaves G. V. Goncalves J. C. Ramalho A. Coelho H. S. (2011). Vitamin A deficiency in patients with hepatitis C virus-related chronic liver disease. Br. J. Nutr. 106 (11), 1724–1731. 10.1017/S0007114511002145 21736776

[B47] Qi F. Inagaki Y. Gao B. Cui X. Xu H. Kokudo N. (2011). Bufalin and cinobufagin induce apoptosis of human hepatocellular carcinoma cells *via* Fas- and mitochondria-mediated pathways. Cancer Sci. 102 (5), 951–958. 10.1111/j.1349-7006.2011.01900.x 21288283 PMC11158054

[B75] Rashid M. M. Varghese R. S. Ding Y. Ressom H. W. (2023). Biomarker discovery for hepatocellular carcinoma in patients with liver cirrhosis using untargeted metabolomics and lipidomics studies. Metabolites 13 (10), 1047. 10.3390/metabo13101047 37887372 PMC10608999

[B48] Somani S. Menta L. Wang-Johanning F. Johanning G. Hong S. Njoo E. (2025). Synthesis, anticancer properties, and biological profiling of synthetic glycan analogs of proscillaridin A. Discov. Pharm. Sci. 1 (1), 25. 10.1007/s44395-025-00025-1

[B76] Sun Q. Zhao W. Wang L. Guo F. Song D. Zhang Q. (2019). Integration of metabolomic and transcriptomic profiles to identify biomarkers in serum of lung cancer. J. Cell. Biochem. 120 (7), 11981–11989. 10.1002/jcb.28482 30805978

[B49] Sung H. Ferlay J. Siegel R. L. Laversanne M. Soerjomataram I. Jemal A. (2021). Global cancer statistics 2020: GLOBOCAN estimates of incidence and mortality worldwide for 36 cancers in 185 countries. Ca. Cancer. J. Clin. 71 (3), 209–249. 10.3322/caac.21660 33538338

[B50] Surovtseva Y. V. Jairam V. Salem A. F. Sundaram R. K. Bindra R. S. Herzon S. B. (2016). Characterization of cardiac glycoside natural products as potent inhibitors of DNA double-strand break repair by a whole-cell double immunofluorescence assay. J. Am. Chem. Soc. 138 (11), 3844–3855. 10.1021/jacs.6b00162 26927829 PMC5530877

[B94] Wang B. Chen D. Chen Y. Hu Z. Cao M. Xie Q. (2012). Metabonomic profiles discriminate hepatocellular carcinoma from liver cirrhosis by ultraperformance liquid chromatography-mass spectrometry. J. Proteome Res. 11 (2), 1217–1227. 10.1021/pr2009252 22200553

[B51] Wang W. Subramani S. (2017). Role of PEX5 ubiquitination in maintaining peroxisome dynamics and homeostasis. Cell. Cycle 16 (21), 2037–2045. 10.1080/15384101.2017.1376149 28933989 PMC5731411

[B52] Waterham H. R. Ferdinandusse S. Wanders R. J. (2016). Human disorders of peroxisome metabolism and biogenesis. Biochim. Biophys. Acta 1863 (5), 922–933. 10.1016/j.bbamcr.2015.11.015 26611709

[B53] Wen J. Xiong K. Aili A. Wang H. Zhu Y. Yu Z. (2020). PEX5, a novel target of microRNA-31-5p, increases radioresistance in hepatocellular carcinoma by activating wnt/Beta-Catenin signaling and homologous recombination. Theranostics 10 (12), 5322–5340. 10.7150/thno.42371 32373215 PMC7196300

[B68] Wen J. Wang Z. Luo S. Aili A. Zou S. Niu S. (2026). Analysis and validation of abnormal signaling pathways and immune cell infiltration characteristics in digestive system cancers based on peroxisome-related genes. Biol. Direct 21 (1), 159. 10.1186/s13062-026-00861-w 42298691 PMC13536876

[B69] Wong T. L. Kong Y. Ma S. (2025). Lipid metabolism in cancer cells: its role in hepatocellular carcinoma progression and therapeutic resistance. Hepatol. Commun. 9 (11), e0837. 10.1097/HC9.0000000000000837 41118283 PMC12548999

[B54] Wu Y. Pi D. Chen Y. Zuo Q. Zhou S. Ouyang M. (2022). Ginsenoside Rh4 inhibits colorectal cancer cell proliferation by inducing ferroptosis *via* autophagy activation. Evidence-based Complementary Alternative Medicine eCAM. 2022, 13–19. 10.1155/2022/6177553

[B55] Wu Y. Pi D. Zhou S. Yi Z. Dong Y. Wang W. (2023). Ginsenoside Rh3 induces pyroptosis and ferroptosis through the Stat3/p53/NRF2 axis in colorectal cancer cells. Acta Biochim. Biophys. Sin. 55 (4), 587–600. 10.3724/abbs.2023068 37092860 PMC10195137

[B56] Xiaolu Wei N. S. Y. Z. Si N. Zhang Y. Zhao H. Yang J. Wang H. (2017). Evaluation of bufadienolides as the main antitumor components in cinobufacin injection for liver and gastric cancer therapy. PLoS One 12 (1), e0169141. 10.1371/journal.pone.0169141 28081155 PMC5231367

[B57] Yang A. L. Wu Q. Hu Z. D. Wang S. P. Tao Y. F. Wang A. M. (2021). A network pharmacology approach to investigate the anticancer mechanism of cinobufagin against hepatocellular carcinoma *via* downregulation of EGFR-CDK2 signaling. Toxicol. Appl. Pharmacol. 431, 115739. 10.1016/j.taap.2021.115739 34619160

[B98] Yang A. Wu Q. Chen Q. Yang J. Li H. Tao Y. (2022). Cinobufagin restrains the growth and triggers DNA damage of human hepatocellular carcinoma cells *via* proteasome-dependent degradation of thymidylate synthase. Chem. Biol. Interact. 360, 109938. 10.1016/j.cbi.2022.109938 35427566

[B59] Yang A. Wu Q. Wang A. Chen Q. Yang J. Tao Y. (2023). Integrated transcriptomics and metabolomics analyses to investigate the anticancer mechanisms of cinobufagin against liver cancer through interfering with lipid, amino acid, carbohydrate, and nucleotide metabolism. Bioorg. Chem. 130, 106229. 10.1016/j.bioorg.2022.106229 36335648

[B60] Yuan B. He J. Kisoh K. Hayashi H. Tanaka S. Si N. (2016). Effects of active bufadienolide compounds on human cancer cells and CD4+CD25+Foxp3+ regulatory T cells in mitogen-activated human peripheral blood mononuclear cells. Oncol. Rep. 36 (3), 1377–1384. 10.3892/or.2016.4946 27431260

[B91] Zhang D. Jiang F. Hu Z. Li A. Wang N. Gao X. (2018). Preventive measures for primary liver cancer. Chin. J. Gerontology 38 (17), 4317–4319.

[B70] Zhang J. Geng W. Sun H. Chen J. Fan Y. Tan K. (2025). Identification of ferroptosis-related genes as diagnostic biomarkers and therapeutic targets in sorafenib-resistant HCC via machine learning and experimental validation. Int. Immunopharmacol. 166, 115557. 10.1016/j.intimp.2025.115557 40983050

[B71] Zhang J. Sun M. Li R. Liu S. Mao J. Huang Y. (2013). Ech1 is a potent suppressor of lymphatic metastasis in hepatocarcinoma. Biomed. Pharmacother. 67, 557–560. 10.1016/j.biopha.2013.03.018 23809371

